# Acute inhibition of acid sensing ion channel 1a after spinal cord injury selectively affects excitatory synaptic transmission, but not intrinsic membrane properties, in deep dorsal horn interneurons

**DOI:** 10.1371/journal.pone.0289053

**Published:** 2023-11-08

**Authors:** Victoria S. Foster, Natalie Saez, Glenn F. King, Michelle M. Rank

**Affiliations:** 1 Institute for Molecular Bioscience, The University of Queensland, St Lucia, Queensland, Australia; 2 St George’s, University of London, Medical School, London, England; 3 Australian Research Council Centre of Excellence for Innovations in Peptide and Protein Science, The University of Queensland, St Lucia, Queensland, Australia; 4 Department of Anatomy and Physiology, School of Biomedical Science, Faculty of Medicine, Dentistry and Health Sciences, The University of Melbourne, Melbourne, Victoria, Australia; University of Missouri-Kansas City, UNITED STATES

## Abstract

Following a spinal cord injury (SCI), secondary damage mechanisms are triggered that cause inflammation and cell death. A key component of this secondary damage is a reduction in local blood flow that initiates a well-characterised ischemic cascade. Downstream hypoxia and acidosis activate acid sensing ion channel 1a (ASIC1a) to trigger cell death. We recently showed that administration of a potent venom-derived inhibitor of ASIC1a, Hi1a, leads to tissue sparing and improved functional recovery when delivered up to 8 h after ischemic stroke. Here, we use whole-cell patch-clamp electrophysiology in a spinal cord slice preparation to assess the effect of acute ASIC1a inhibition, via a single dose of Hi1a, on intrinsic membrane properties and excitatory synaptic transmission long-term after a spinal cord hemisection injury. We focus on a population of interneurons (INs) in the deep dorsal horn (DDH) that play a key role in relaying sensory information to downstream motoneurons. DDH INs in mice treated with Hi1a 1 h after a spinal cord hemisection showed no change in active or passive intrinsic membrane properties measured 4 weeks after SCI. DDH INs, however, exhibit significant changes in the kinetics of spontaneous excitatory postsynaptic currents after a single dose of Hi1a, when compared to naive animals (unlike SCI mice). Our data suggest that acute ASIC1a inhibition exerts selective effects on excitatory synaptic transmission in DDH INs after SCI via specific ligand-gated receptor channels, and has no effect on other voltage-activated channels long-term after SCI.

## Introduction

Acidification of the central nervous system (CNS) microenvironment is a key mechanism of secondary damage within the CNS. Vascular damage after a spinal cord injury (SCI) causes a marked reduction in blood flow to tissue surrounding the lesion. Neurons and glia in the center of the ischemic territory, where blood flow is lowest, are rapidly and irreparably damaged. However, at the periphery of this area of primary damage, the peri-injury region, collateral vessels maintain perfusion above the threshold for immediate cell death [1] and thus a cascade of secondary damage evolves over a period of hours to days. In the absence of sufficient blood flow, the resulting hypoxia in the peri-injury region forces neurons to function in an anaerobic state, which in turn causes a build-up of extracellular H^+^ and acidification in the region (tissue acidosis).

Acid-sensing ion channels (ASICs) detect tissue acidosis. ASICs are a family of proton-gated ion channels that are permeable to Na^+^ and activated by H^+^ [2]. After ischemic stroke or SCI, downstream hypoxia and acidosis in the brain or spinal cord activate ASIC1a, a receptor subtype that is highly expressed in the CNS (as well as immune cells) and is additionally permeable to Ca^2+^ [3–7]. In the brain, this triggers RIP1-kinase-mediated necroptotic cell death [8]. Increased ASIC1a expression has been shown to occur 3–24 h after traumatic SCI in the peri-injury zone with a converse decrease in expression at the lesion epicentre [9]. Knockout or knockdown of ASIC1a expression and specific pharmacological inhibition of ASIC1a are neuroprotective, leading to tissue sparing long-term after SCI and ischemic stroke [9–14]. However, pan-ASIC inhibition using amiloride, or ablation of the *ASIC1* gene, has been reported to enhance excitotoxic cell death in an *in vitro* model of SCI [15]. Although the role of ASIC1a in brain plasticity has been robustly investigated, the effect of ASIC1a inhibition on neuronal transmission in the uninjured spinal cord, or after SCI, remains unknown [16].

A critical population of interneurons (INs) located in the deep dorsal horn (DDH) of the spinal cord (laminae III-V) carry out the processing of somatosensory input and, importantly, act to shape motor output [17–19]. In rodents, descending corticospinal tract inputs also terminate in the DDH [17, 20], meaning that DDH INs serve as the all-important ‘link’ between upper and lower motoneurons. Indeed, alterations in the intrinsic membrane properties and excitatory synaptic transmission of DDH IN populations, measured via whole-cell patch clamp electrophysiology, have been shown to correlate with motor improvements after SCI [21]. DDH INs have additionally been shown to be responsive to interventions, such as exercise training, demonstrating long-term alterations to membrane conductances and synaptic transmission up to 9 weeks after SCI [22–24]. Electrophysiological readouts from this unique spinal cord IN population are thus a robust measure of post-SCI neuronal plasticity in spinal cord motor circuits. How this critical population of INs responds to a neuroprotective treatment, such as ASIC1a inhibition, has not yet been investigated.

We hypothesised that limiting ischemia-induced activation of neuronal ASIC1a after SCI might minimise the extent of secondary damage and improve neurological outcomes, as measured by whole-cell patch-clamp electrophysiology of DDH INs in a mouse spinal cord slice preparation. Currently the most potent inhibitor of ASIC1a is Hi1a, a recently discovered double-knot venom peptide. Hi1a inhibits both human and rat ASIC1a with an IC_50_ of ~500 pM [12]). Here we used a single moderate dose of Hi1a (25 μg, i.v.), administered acutely after a spinal cord hemisection (1 h post-SCI), to probe the effects of ASIC1a inhibition on long-term changes to intrinsic membrane and excitatory synaptic transmission properties of DDH INs. This study provides, for the first time, a direct and detailed readout of *functional* properties of spinal cord INs after SCI and acute ASIC1a inhibition.

## Materials and methods

### Mouse hemisection model of SCI

All procedures performed for these experiments were approved by the RMIT University Animal Ethics Committee (Approval number AEC1710). Animals were obtained from RMIT University Research Animal Facility. The study was not pre-registered. Mice with incomplete SCI (lateral hemisection) were used for this study. This injury results in ipsilateral hindlimb paralysis post-surgery. Since the contralateral leg remains functional, mice exhibit normal behaviour in the recovery period after surgery. Mice can be housed together within days, and thus experience normal social interactions. Hemisection (as opposed to contusion) SCI is preferred for electrophysiological studies as it provides viable tissue in the peri-injury region to assess the electrophysiological properties of INs.

The procedures for spinal cord hemisection surgery have been described previously [21–26]. The timeline for the following procedure and subsequent experiment is shown in S1 Fig. Briefly, a left T10 spinal cord hemisection (between T10 and T11 spinal nerves) was administered to adult male C57/Bl6 mice (RRID:IMSR_JAX:000664; aged 9–10 weeks) under isoflurane anaesthesia, allowing mice to awaken shortly after surgeries to be monitored. A single experienced surgeon performed all surgeries to control for procedural variability. No randomisation of animals was performed. During each surgery, the depth of anaesthesia and body temperature were monitored and recorded at regular intervals. To minimise animal suffering, we employed a number of analgesics for treatment post-surgery. Firstly, post-surgical analgesia was provided by subcutaneous (s.c.) buprenorphine (0.1 mg/kg) and carprofen (5 mg/kg) administered immediately after anaesthesia induction at the beginning of each surgery. Additional post-surgical analgesia was provided by carprofen (5 mg/kg) every 12 h for 48 h, and a 1 cm^2^ fentanyl patch (Duragesic 12 mcg/h) placed on the shoulder which delivered steady analgesia for 72 h post-surgery. Mice also received s.c. saline injections every 12 h for 72 h, delivering hydration support. Nutritional support was achieved by providing mash (soaked chow pellets) and nutritional gels until normal feeding behaviour returned, usually at 48–72 h post-surgery. The fentanyl patch was removed after 72 h of recovery in isolated cages, then mice were returned to their normal home cage groups of 2–4 mice. Mice were assessed for motor function at 3 days post-surgery. Exclusion criteria were applied to mice that exhibited paralysis of both hindlimbs; they were not included in the study and were instead humanely euthanised using an intraperitoneal (i.p.) injection of sodium pentobarbitone (100 mg/kg, Virbac; *n* = 4). Mice were allowed to recover for four weeks post-surgery and then humanely euthanised as described below for whole-cell patch-clamp electrophysiology experiments.

Mice were split into three experimental cohorts for this study: naive, SCI, and SCI + Hi1a (Table 1). Naive mice received no surgery (i.e., were uninjured) and received no treatment intervention. SCI and SCI + Hi1a groups both underwent identical spinal cord hemisection surgery. The SCI + Hi1a group additionally received acute treatment with the ASIC1a inhibitor Hi1a (25 μg i.v.) via tail vein injection 1 h after SCI surgery. Following the rules of reduction, refinement, and replacement (RRR), and due to the level of technical challenge for these experiments, we did not include a sham surgery group. Recombinant Hi1a was produced as described previously [12]. Due to the nature of the data in which no parameters can be modified by the experimenter, no blinding was performed.

**Table 1:** Treatment and experimental group numbers.

Treatment group	Experimental Numbers		
	Electrophysiology experiments (# of mice)	Intrinsic properties (# of INs)	Synaptic properties (# of INs)
Naive	36	36	25
Control SCI	17	20	24
SCI + Hi1a *(25 μg*, *i*.*v*.*)*	14	30	27

Statistical power was calculated using G*Power 3.1 [27] using α = 0.05. Tissue was collected from: (i) Naive mice (no intervention); (ii) SCI mice; and (iii) SCI + Hi1a mice (25 μg Hi1a in 0.2 ml of saline i.v.). The total number of mice allocated to each cohort is indicated. For intrinsic membrane and synaptic properties, the number of DDH INs recorded using whole-cell patch-clamp electrophysiology is indicated.

Electrophysiological recordings were made from naive mice at 8–13 weeks of age, whereas SCI and SCI + Hi1a cohorts were 12–13 weeks old at the time of electrophysiology experiments. Experiments were performed first on naive animals, and subsequently the SCI cohorts. Earlier research showed no difference in outcome between these age groups [23, 24]. Mice were humanely euthanised as described below, for *in vitro* whole-cell patch-clamp electrophysiology 4 weeks after the SCI surgery or, for naive mice, at 12–13 weeks of age. This was the primary endpoint of the experiment. A unique spinal cord slice preparation was used to perform whole-cell patch-clamp electrophysiology experiments to examine the effect of Hi1a on DDH IN function (S1B Fig). The spinal cord was prepared using a method previously described [22, 28]. Mice were deeply anaesthetised using ketamine (100 mg/kg i.p.) and transcardially perfused with ice-cold, oxygenated, sucrose artificial cerebrospinal fluid (sACSF containing in mM: 250 sucrose, 25 NaHCO_3_, 11 glucose, 2.5 KCl, 1 NaH_2_PO_4_, 6 MgCl_2_, and 1 CaCl_2_; pH 7.3–7.4). The animal was then decapitated, eviscerated, and the torso isolated and submerged in ice-cold sACSF. The spinal cord was carefully extracted using a ventral approach, then the T5–T13 section of the cord was isolated. Note that this spinal cord segment fully encompasses the T10 lesion site including the peri-injury region (S1B Fig). The section was then mounted on agar (6%) on the cutting stage with cyanoacrylate glue. Using a vibratome (HM 650 V; Microm, Walldorf, Germany or VT1200s, Leica Microsystems, Nuslock, Germany), a single 250-μM thick horizontal slice was taken that includes the dorsal horn, intermediate zone, and a continuous strip of the dorsal columns containing the corticospinal tract. The spinal cord slice was placed under a custom-made platinum and nylon net and equilibrated for 1 h at room temperature (22–25°C) prior to recording, a procedure used extensively by our group and others [21–23, 28, 29]. As bath temperature is known to modify specific intrinsic neuronal properties (Graham, Brichta [30]), temperature in the bath/recording chamber was maintained constant via an in-line heater paired to a temperature probe in the bath and set to maintain the bath temperature at 23–24°C via a feedback loop.

Throughout the equilibration period and continuously during electrophysiology experiments, the spinal cord slice was perfused with ACSF oxygenated with medical carbogen (95% O_2_ and 5% CO_2_ resulting in a pH of 7.3–7.4), containing (in mM): 118 NaCl, 25 NaHCO_3_, 11 glucose, 2.5 KCl, 1 NaH_2_PO_4_, 1 MgCl_2_, and 2.5 CaCl_2_.

### Whole-cell patch-clamp electrophysiology

**Fig 1:**
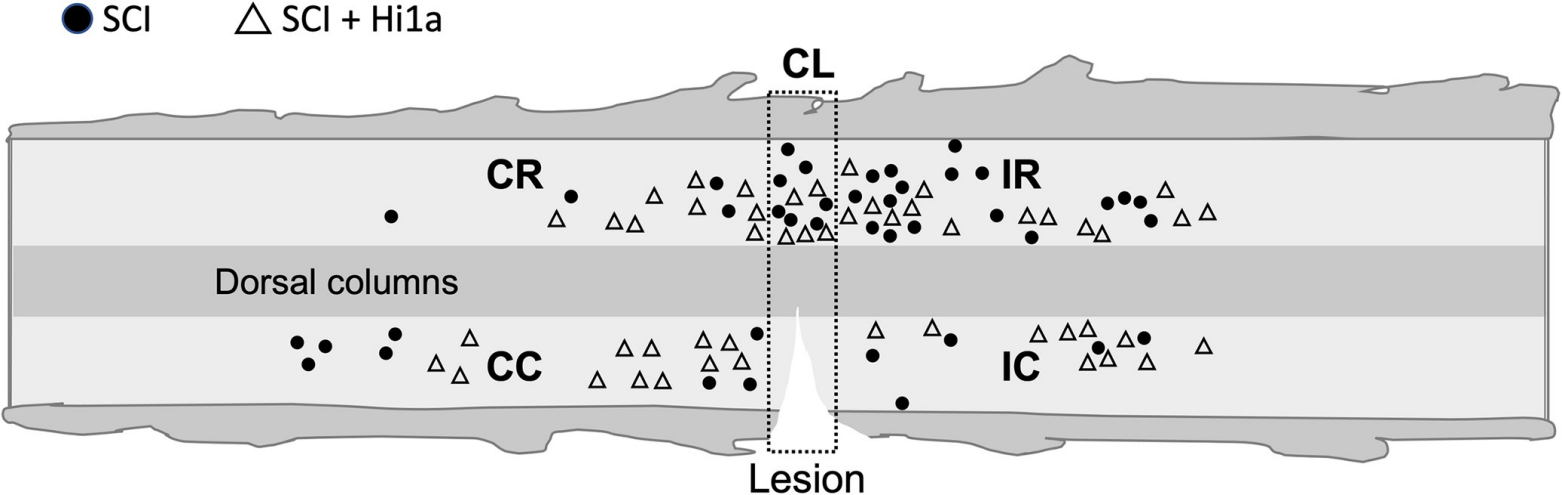
Location of recorded DDH INs from SCI experimental groups. Schematic with approximate location of recorded DDH INs in the vicinity of a SCI on a horizontal slice, based on tiled image mapping. Lighter areas of shading represent the grey matter and darker areas for white matter, which are well defined under infrared differential interference contrast (IR-DIC) optics. INs from untreated SCI group (filled circle) and SCI + Hi1a treatment group (open triangle) were recorded using whole-cell patch-clamp electrophysiology at a maximum of two spinal cord segments from the lesion epicentre. The spinal cord slice was divided into five location-based regions: contralateral caudal (CC); contralateral rostral (CR); ipsilateral caudal (IC); ipsilateral rostral (IR) and contralateral to the lesion epicentre (CL) (within dashed rectangle, at T10).

All experiments were performed at room temperature, and between 8 am and 8 pm. Whole-cell patch-clamp electrophysiology recording procedures were based on protocols previously described by Rank, Flynn [23]. Briefly, recordings were made using an Axopatch 200B amplifier (Molecular Devices, Sunnyvale, USA). Borosilicate glass patch pipettes (1.5 mm OD × 1.16 mm ID; Harvard Apparatus, Kent, UK) were pulled to a tip resistance of 3–5 MΩ and filled with a K^+^-gluconate internal solution, pH 7.2, 290–300 mOsm/kg (in mM: 135 KCH_3_SO_4_, 6 NaCl, 2 MgCl_2_, 10 HEPES, 0.1 EGTA, 2 MgATP, and 0.3 NaGTP). Data were recorded using AxoGraph X software (AxoGraph Scientific, Sydney, Australia), using a sampling rate of 50 kHz with 2–10 kHz filtering. DDH INs were targeted for recording using infrared differential interference contrast (DIC) optics. Prior to recording, photographs of the entire horizontal slice were taken in a sequence of images using an Olympus DP50 digital camera and Viewfinder lite software (Olympus, Tokyo, Japan). After each recording was complete, the location of the DDH IN was photographed with the patch pipette still in contact. Using an averaged template of the spinal cord slice, DDH IN locations were plotted on the schematic template (see Fig 1), ensuring that they were all within two spinal segments of the lesion.

Passive and active intrinsic membrane properties were recorded from DDH INs as follows: resting membrane potential (RMP), input resistance (I_R_), rheobase current, action potential (AP) threshold, and after-hyperpolarisation (AHP) amplitude. AP firing properties such as discharge type, AP peak and width were also analysed. Excitatory synaptic transmission was analysed from recordings of spontaneous excitatory postsynaptic currents (sEPSCs). Finally, subthreshold currents were activated and identified using specific ramp protocols as described below.

### Electrophysiological recordings

**Fig 2:**
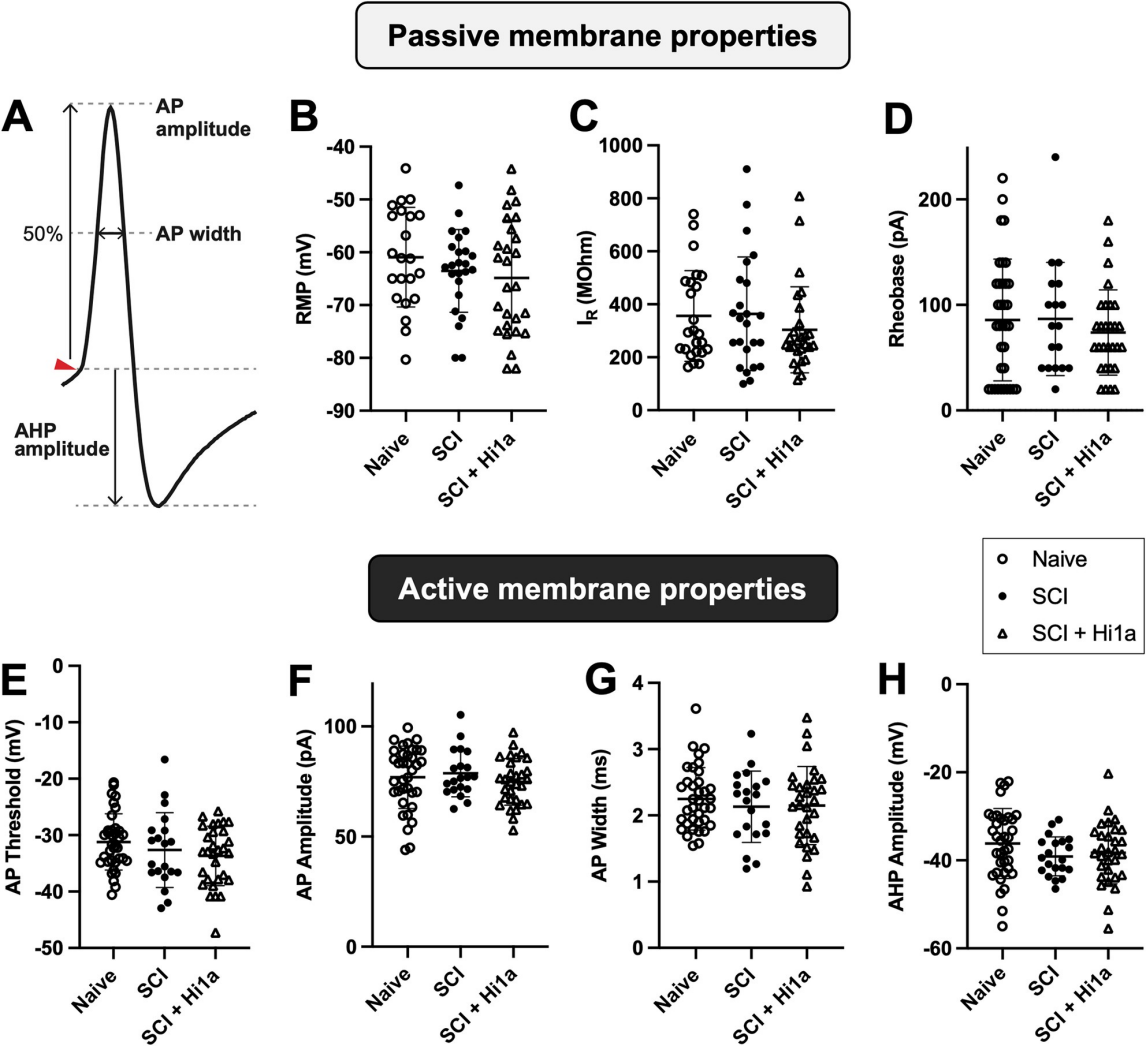
Passive and active membrane properties of DDH INs in naive, SCI, and SCI + Hi1a treated mice. Individual values are shown, with mean ± SD. (**A**) Schematic to demonstrate active membrane properties, as measured through APs (AP amplitude, AP width and AHP amplitude). **B–D**: Passive membrane properties. (**B**) RMP for DDH INs for naive uninjured mice (“Naive”; open circles, n = 22 neurons), SCI mice (“SCI”; filled circles, n = 24), and SCI mice treated with Hi1a (“SCI + Hi1a”; open triangle, n = 27), (**C**) I_R_ for naive (n = 24), SCI (n = 24) and SCI + Hi1a (n = 27) (**D**) rheobase for naive (n = 35), SCI (n = 18) and SCI + Hi1a (n = 29). **E–H**: Active membrane properties (**E**) AP properties in spinal cord DDH INs of naive, SCI and SCI + Hi1a mice. (**F**) AP threshold for neurons from naive (n = 36 neurons), SCI (n = 20), and SCI + Hi1a (n = 30). (f) AP amplitude for naive (n = 36), SCI (n = 20) and SCI + Hi1a (n = 30). (**G**) AP width for naive (n = 36), SCI (n = 20) and SCI + Hi1a (n = 30). (**H**) AHP amplitude for naive (n = 36), SCI (n = 20) and SCI + Hi1a (n = 30). NS: Significance tested using ordinary one-way ANOVA with Tukey’s multiple comparisons test (**B, D, E, G** and **H**) or Kruskal–Wallis non-parametric one-way ANOVA with Dunn’s multiple comparisons (**C** and **F**).

The whole-cell recording configuration was initially established in voltage-clamp mode (holding potential –60 mV; series (access) resistance < 26 MΩ) [29, 31, 32]. Holding potential is consistent with the average RMP seen for INs in all cohorts (Fig 2A). A 5 mV square hyperpolarising step (10 ms duration, 10 repetitions) was used to monitor series and input resistance. All recordings were adjusted by –10 mV to adjust for the calculated liquid junction potential (LJP) between the ACSF and internal patch pipette solution [33].

Only INs with a stable I_R_ of above 100 MΩ were included for analysis, as lower values reflect adverse cellular conditions [34]. Real-time measures of I_R_ were monitored using the in-built test pulse window in the data acquisition software (AxoGraph X). The test-pulse program estimates series resistance and membrane resistance by fitting an exponential to the decaying phase of the response to a 5 ms –10 mV pulse step in voltage-clamp mode. The accuracy of series and membrane resistance measures was confirmed manually and calibrated at the onset of each experiment. The number of INs with an access resistance of 20–26 MΩ included for analysis were as follows: naive: n = 5 out of 24, SCI: n = 6 out of 24 and SCI + Hi1a: n = 8 out of 27. The series resistance values for naive (mean ± SD: 18.8 ± 3.7 MΩ), SCI (19.8 ± 3.3 MΩ) and SCI + Hi1a (17.8 ± 4.2 MΩ) INs showed overall only a small deviation from 20 MΩ. However, as access >20 MΩ has been proposed to reflect poor electrical access and insufficient voltage clamp of the membrane, Spearman’s r correlation statistics were performed [34]. This revealed no significant difference between any parameters measured when INs with a series resistance of higher than 20 MΩ were included (S5 Table). This mixed population of DDH INs have complex dendritic arbours that likely extend across several spinal segments. Ensuring adequate space clamp of electrotonically distant synapses is technically very challenging. Although we cannot ensure that our space clamp was faultless, all recordings were made under similar conditions and therefore any issues associated with poor space clamp in dendrites is expected to be similar across all preparations.

AP properties and discharge characteristics were examined by injecting square current pulses (20 pA increments, 800 ms duration). Individual APs were captured using a derivative threshold method, with the threshold set at a d*V*/d*t* value between 10 and 15 V/s. Rheobase current was defined as the smallest step current that elicited at least one AP. AP threshold, peak amplitude (measured from point of inflection to peak), and afterhyperpolarisation (AHP) peak (measured from point of AHP inflection to negative peak) were quantified from rheobase APs.

**Fig 3:**
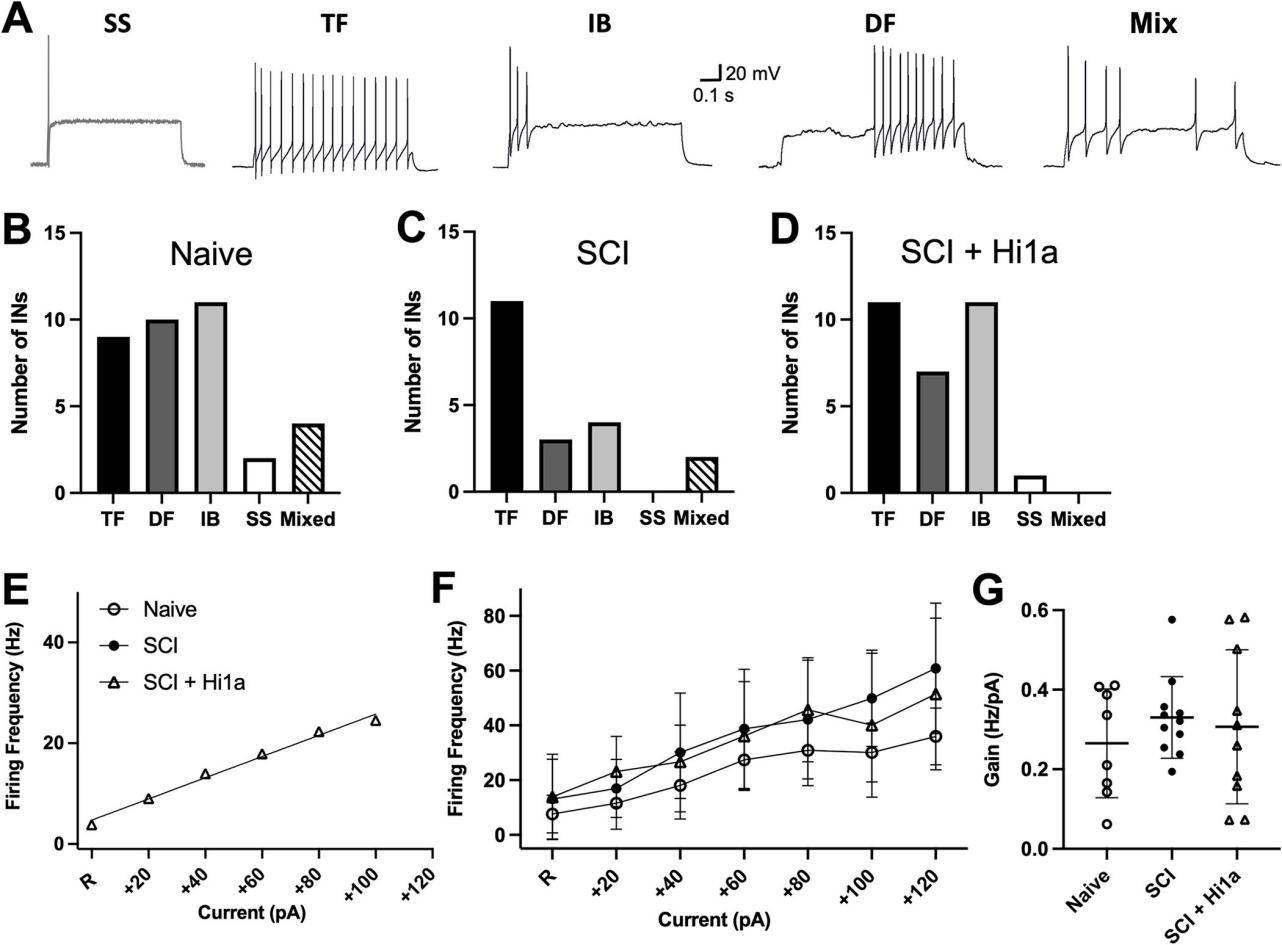
AP discharge patterns. (**A**) The four main types of AP discharge patterns: single spiking (“SS”); tonic firing (“TF”); initial bursting (“IB”), delayed firing (“DF”) and mixed (“mix”). (**B–D**) Prevalence of AP discharge patterns for DDH INs in (**B**) naive uninjured mice (“Naive”) for TF (black, n = 9 neurons), DF (dark grey, n = 10), IB (light grey, n = 11), SS (white, n = 2) and mix (striped, n = 4); (**C)** SCI mice (“SCI”) for TF (black, n = 11 neurons), DF (dark grey, n = 3), IB (light grey, n = 4), SS (white, n = 0) and mix (striped, n = 2); (**D**) SCI mice treated with Hi1a (“SCI + Hi1a”) for TF (black, n = 11 neurons), DF (dark grey, n = 7), IB (light grey, n = 11), SS (white, n = 1) and mix (striped, n = 0). NS: significance tested using two-tailed Fisher’s exact tests. (**E**) Sample plot from single TF IN, showing linear slope. (**F**) F-I plots for DDH INs displaying the tonic firing (TF) discharge pattern plot from rheobase (R) to +120 pA for DDH INs for naive uninjured (open circle, n = 8), SCI (filled circle, n = 10), and SCI + Hi1a (open triangle, n = 11). Mean ± SD, NS: Significance tested using mixed effects analyses. (**G**) Amplification properties (gain) calculated from F-I plots, with mean ± SD (between rheobase up to +120 pA) for DDH INs for naive uninjured (n = 8), SCI (n = 10), and SCI + Hi1a (n = 11).

We documented the incidence of discharge patterns across each experimental group as these properties reflect specific ion channel expression patterns [35]. AP discharge patterns were classified into four specific patterns, as previously reported [29, 35, 36]. Single spiking (SS) INs discharge a single AP at each current step; tonic firing (TF) INs discharge a train of APs; initial bursting (IB) INs discharge 2–5 APs at the beginning of the current step; and delayed firing (DF) INs discharge APs only towards the end of the step. INs that could not be clearly classified into one of these groups were classified as ‘mixed’ (Fig 3A).

The frequency-current (F-I) relationship for INs that displayed TF discharge patterns was also examined. To determine the F-I relationship, the AP frequencies were averaged and plotted against each current step increment, with the slope of the line indicating the F-I gain. F-I plots for individual cells consistently displayed linear relationships; the slope was calculated for individual cells (Fig 3E) before aggregating the data (Fig 3F). The gain of the F-I plot reflects the amplification properties of DDH INs.

The expression of several voltage-gated ion channels is known to underlie different AP discharge patterns in mouse dorsal horn INs [29, 37, 38]. For example, hyperpolarisation-activated cyclic nucleotide-gated (HCN) channels such as HCN1, 2 and 4 contribute to fast spiking patterns in mice [39, 40]. Specific ablation of Ca_V_3.2 channels alters neuron firing type in the mouse dorsal horn to mainly TF and DF patterns [41]. Potassium currents in the dorsal horn of the mouse have been shown to be driven by rapid outward K^+^ A-current (I_Ar_), slow outward K^+^ A-current (I_As_) and low-threshold activated T-type Ca^2+^ current (Ca_V_3, I_Ca_) [42].

To investigate the prevalence of different subthreshold currents we applied a specific voltage-clamp protocol from a holding potential of –60 mV to evoke subthreshold currents [29]. A hyperpolarising pulse to –90 mV (1 s duration) was delivered followed by a depolarising step to −40 mV (200 ms duration) (Fig 5A). Automated P/N leak subtraction was used to remove capacitive and leakage currents. There are three main types of voltage-activated subthreshold currents evoked by this protocol: I_Ar_; I_As_; and I_Ca_ [42].

Spontaneous excitatory synaptic currents (sEPSCs) were recorded for a minimum of 180 s using a holding potential of –60 mV and analysed offline. ASIC1a has been shown to contribute to postsynaptic signalling and is present in all regions of the spinal cord, including dorsal horn neurons in both rats and mice, with the mouse isoform bearing 98% similarity to the human channel [43–49]. Previous work has shown that under these conditions in this model, sEPSCs are exhibited as inward currents (see example trace in Fig 5A–5C) driven by α-amino-3-hydroxy-5-methyl-4-isoxazolepropionic acid receptors (AMPARs), as the majority are eliminated by addition of 10 μM of the AMPAR antagonist CNQX [28].

sEPSC frequency was calculated from captured events using a template in AxoGraph to detect sEPSCs between 5–120 pA (excluding noise and APs) over 180 s of recording. Manual screening was also performed to filter noise captured by the template. The captured and filtered sEPSCs were averaged to acquire peak amplitude (baseline to current peak), rise time (measured from 10–90% of peak amplitude), half-width (calculated at 50% of peak amplitude), and decay time constant (calculated over 20–80% of the decay phase).

### Statistical analyses

Data distribution and normality were assessed using Kolmogorov–Smirnov and Shapiro–Wilk tests. Since our experimental design includes multiple comparisons across three experimental groups, the use of multiple *t*-tests to undertake these comparisons increases the possibility of statistical error [50]. Therefore, parametric data with a normal distribution were analysed using an ordinary one-way analysis of variance (ANOVA), followed by Tukey’s multiple comparison post-hoc tests. Non-parametric data were analysed by Kruskal–Wallis one-way ANOVA with Dunn’s multiple comparisons post-hoc tests. χ2 and two-tailed Fisher’s exact tests were used to compare nominal data (AP discharge categories and subthreshold current types). To evaluate the impact of series resistance and RMP variability on reported values we performed two-tailed Spearman’s r correlation analyses (S5 and S6 Tables) which indicated that neither series resistance nor RMP had a significant effect on the membrane or sEPSC parameters measured. Relative and cumulative frequency of sEPSC rise time data were binned (0.3 ms and 0.2 ms bins, respectively) and the bins were compared using a two-way ANOVA and Tukey’s multiple comparisons post-hoc tests. Significance for all statistical analyses was set at p < 0.05. All statistical analyses were performed using Prism 7 (GraphPad Software Inc., La Jolla, USA). Results are reported as mean ± standard deviation (SD). Statistical power was calculated using G*Power 3.1 [27] using α = 0.05 and a moderate effect size (*d* = 0.7), determined from previously reported membrane and synaptic properties [21–23].

## Results and discussion

We examined selected intrinsic properties in DDH INs following incomplete SCI in mice treated, via a single intravenous injection, with the ASIC1a inhibitor Hi1a 1 h after surgery. The same intrinsic properties were also recorded in DDH INs from SCI mice and uninjured naive mice. Data were obtained from neurons located within two spinal cord segments of the T10 hemisection injury (Fig 1). Values for all results, including those not stated in text, are noted in S1–S6 Tables.

### Proximity of INs to spinal cord lesion

Each spinal cord slice was photographed in its entirety under brightfield conditions with 40-fold magnification while the slice was equilibrating in the recording chamber. Individual images were montaged in PowerPoint. Each recorded neuron was additionally photographed and the location on the spinal cord slice mapped onto a template spinal cord slice schematic (see Fig 1). To determine whether the location of the DDH IN relative to the spinal cord lesion had any effect on the electrophysiological parameters measured in the study, we designated neuron location as one of five areas—(i) contralateral caudal (SCI, n = 3; SCI + Hi1a, n = 4), (ii) contralateral rostral (SCI, n = 7; SCI + Hi1a, n = 11), (iii) ipsilateral caudal (SCI, n = 8; SCI + Hi1a, n = 4), (iv) ipsilateral rostral (SCI, n = 2; SCI + Hi1a, n = 2), and (v) contralateral to epicentre (SCI, n = 2; SCI + Hi1a, n = 2)—and compared areas for each electrophysiological parameter.

IN location had no effect on intrinsic membrane (R_in_, rheobase current and RMP) or active action potential (AP) properties (AP threshold, AP amplitude, AHP amplitude) in SCI or SCI + Hi1a treated mice (S3 Table). There was no effect of IN location for active membrane properties (sEPSC frequency, rise, decay, half-width and peak) in either cohort, other than sEPSC frequency, between the contralateral caudal versus contralateral rostral regions of the spinal cord (Dunn’s multiple comparisons test: p <0.005) for Hi1a-treated animals. We note that grouped mean sEPSC frequency data, from all spinal cord regions, is not significantly different between the SCI and SCI + Hi1a experimental groups. The results we report here are similar to those reported previously [21, 23, 24] and suggest that all neurons in close proximity, at least two spinal segments, of the spinal cord lesion were similarly affected by the spinal cord hemisection injury and acute Hi1a treatment.

### Intrinsic membrane properties in DDH Ins

Passive intrinsic membrane properties (RMP, I_R_ and rheobase) of DDH INs from naive, SCI, and SCI + Hi1a cohorts are shown in Fig 2. There was no significant difference across or between experimental groups for any of the intrinsic membrane properties tested (one-way ANOVA with multiple comparisons), namely RMP (Fig 2B), I_R_ (Fig 2C) and rheobase (Fig 2D). RMPs were slightly hyperpolarised for the SCI (–63.07 mV ± 9.84) and SCI + Hi1a treated (–64.45 mV ± 10.95) mice when compared to naive (–60.7 mV ± 9.43). Taken together, the data indicate that acute inhibition of ASIC1a via single i.v. dose of Hi1a after SCI has no lasting effects on the passive intrinsic membrane properties of DDH INs.

To measure active intrinsic membrane/AP properties, AP current steps ranging from 0–240 pA were injected in current clamp, using a maximum of 13 current steps to evoke APs. Injected current required to elicit threshold APs was not different for naive (mean ± SD: 83.33 ± 58.65 pA), SCI (78.00 ± 57.26 pA) and SCI + Hi1a (71.33 ± 41.91 pA) INs. One-way ANOVA tests with Tukey’s multiple comparisons testing did not reveal any significant differences between the active membrane properties of spinal cord DDH INs in the naive, SCI and SCI + Hi1a experimental groups, including AP threshold (Fig 2E), AP amplitude (Fig 2F), AP width (Fig 2G) and AHP amplitude (Fig 2H). Importantly, analysis using Spearman’s rank correlation revealed no significant effect of RMP on any AP parameters within cohorts (S5 Table).

### AP discharge patterns and TF frequency in DDH INs

To assess the effect of acute single-dose ASIC1a inhibition on long-term changes to specific ion channel conductances of DDH INs, we examined the incidence of AP discharge patterns across the three experimental groups (naive, SCI and SCI + Hi1a). AP discharge patterns were elicited by injecting an 800 ms square-step depolarising current into spinal cord DDH INs from RMP. AP current steps ranged from 0–240 pA, using a maximum of 13 current steps to evoke APs for naive (mean ± SD: 83.33 ± 58.65 pA), SCI (78.00 ± 57.26 pA) and SCI + Hi1a (71.33 ± 41.91 pA). The resulting AP discharge patterns can be categorised into four groups as described previously [21–24]: tonic firing (TF), initial bursting (IB), delayed firing (DF) or single spiking (SS) (Fig 3A). Each of the four discharge categories reflect the expression of specific ion channel conductances and therefore provide a reliable readout of the properties of specific voltage-gated subthreshold currents [29, 37, 38, 51], notably voltage-gated Na^+^ and delayed-rectifier K^+^ currents. SS INs were found to exhibit APs at any point during the current step, but typically at current step onset. We also included a designation here (mixed) for INs that exhibited multiple discharge patterns in response to depolarising current steps, which reflected the expression of multiple simultaneous ion channel conductances (Fig 3A).

All four major discharge patterns (TF, IB, DF and SS) were observed in the experimental groups (naive, SCI and SCI + Hi1a), with the notable exception of the SS discharge category absent from the SCI group. Additionally, the proportion of neurons exhibiting each discharge pattern differed. The relative prevalence of DF and IB discharge patterns is similar across naive and SCI + Hi1a mice when compared to the prevalence of these same discharge patterns in the SCI group (Fig 3B–3D). DDH INs in the SCI group did not exhibit a SS firing pattern, whereas both naive and SCI + Hi1a mice showed a low prevalence of SS firing INs. DDH INs recorded from the SCI group exhibited the TF discharge patterns as the dominant AP firing type, whereas in the naive group, the IB discharge pattern was dominant and, in the SCI + Hi1a group, the highest incidence was split between TF and IB. Although statistically significant differences were not present, some reorganisation of all discharge patterns was observed across the treatment groups. This likely reflects the heterogeneity of the population of INs in the DDH with differences in the expression of specific ion channel conductances in SCI mice, whether or not they were treated acutely with an ASIC1a inhibitor, as compared to naive uninjured mice. When compared using two-tailed Fisher’s exact tests, there were no significant differences in DDH IN AP discharge patterns across naive, SCI and SCI + Hi1a groups.

As the TF discharge pattern is the dominant AP discharge pattern expressed by DDH INs of the SCI group we examined whether acute ASIC1a inhibition via Hi1a treatment affected the firing frequency and gain of INs expressing the TF phenotype (F-I plots; Fig 3F). Firing frequencies are presented as normalised to rheobase as there were no significant differences in rheobase across the naive, SCI and SCI + Hi1a groups (Fig 2D). Data were plotted to a maximum of +120 pA from rheobase. There were no significant differences in firing frequency between groups at any current step.

The modulation of gain in the frequency of AP firing is a central feature of neural information processing [52–54]. We sought to understand whether DDH INs exhibit long-lasting changes in information processing capacity after SCI and acute ASIC1a inhibition by calculating the gain for TF INs in naive, SCI and SCI + Hi1a groups. Gain was measured as the slope of the F-I (input-output) relationship over input currents ranging from rheobase to +120 pA (Fig 3E–3G). Analyses using Kruskal–Wallis non-parametric one-way ANOVA with Dunn’s multiple comparisons demonstrated no significant differences across the experimental groups in the gain of TF INs. Firing frequency tended to be lower in naive animals compared to the SCI groups with no significance observed between groups when mixed effects analyses were performed. This suggests that information processing parameters of DDH INs are changed with SCI alone, but are not differentially affected when SCI animals receive acute treatment with Hi1a.

### Subthreshold currents

**Fig 4:**
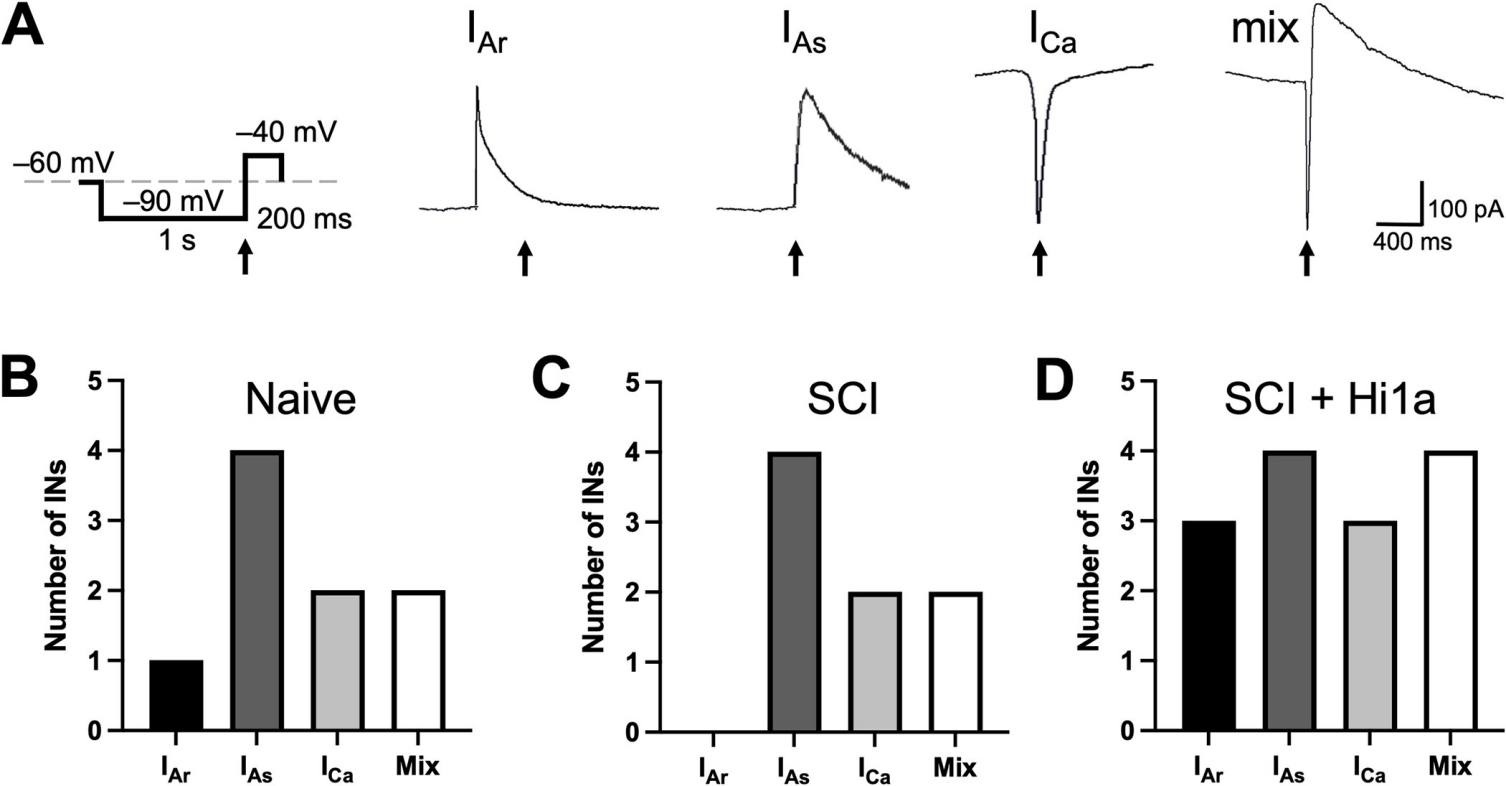
Select voltage-gated subthreshold currents recorded from DDH INs in naive, SCI and SCI + Hi1a treated mice. (**A**) Voltage-step protocol used to elicit subthreshold currents. Grey dashed line represents –60 mV holding current. Representative currents shown for rapid outward K^+^ A-current (“I_Ar_”), slow outward K^+^ A-current (“I_AS_”), low-threshold activated T-type Ca^2+^ current (“I_Ca_”), and a mixed current (“mix”) of both I_Ca_ and I_As,_ respectively. Black arrows showing location of evoked current during protocol (**B–D**) Prevalence of voltage-gated subthreshold currents for DDH INs in (**B**) naive, uninjured mice (“Naive”) for I_Ar_ (black, n = 1 neuron), I_AS_ (dark grey, n = 4), I_Ca_ (light grey, n = 2), and mix (white, n = 2); (**C**) SCI mice (“SCI”) I_Ar_ (black, n = 0 neurons), I_As_ (dark grey, n = 4), I_Ca_ (light grey, n = 2), and mix (white, n = 2) and (**D**) SCI mice treated with Hi1a (“SCI + Hi1a”) for I_Ar_ (black, n = 3 neurons), I_As_ (dark grey, n = 4), I_Ca_ (light grey, n = 3), and mix (white, n = 4). NS: significance tested using two-tailed Fisher’s exact tests.

**Fig 5:**
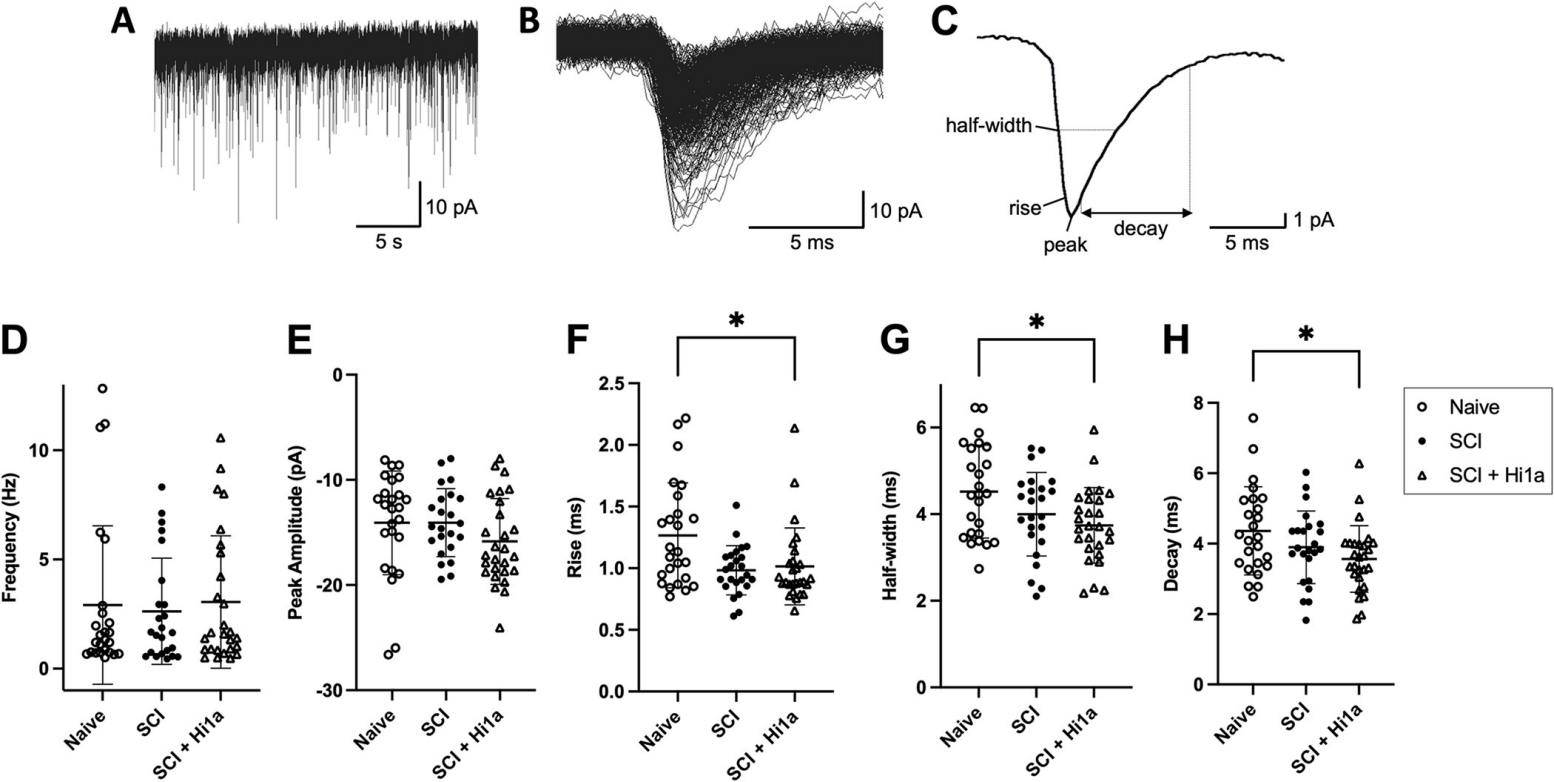
sEPSC frequency and current kinetics in DDH INs of naive, SCI and SCI + Hi1a mice. For all plots, each marker indicates averaged data from an individual IN and horizontal lines indicate mean ± SD. (**A**) Raw trace of sEPSCs recorded in voltage-clamp mode from –60 mV holding potential. (**B**) Individual sEPSCs isolated from a raw trace and overlaid. (**C**) Average of isolated sEPSCs demonstrating measured sEPSC properties (average half-width, rise, peak and decay). (**D**) Average sEPSC frequency for DDH INs from naive uninjured mice (“Naive”; open circles, n = 25 neurons), SCI mice (“SCI”; filled circles, n = 24), and SCI mice treated with Hi1a (“SCI + Hi1a”; open triangle, n = 27). (**E**) Average peak amplitude for naive (n = 25), SCI (n = 24) and SCI + Hi1a (n = 27). (**F**) Average rise time for naive (n = 25), SCI (n = 24) and SCI + Hi1a (n = 27). (**G**) Average half-width for naive (n = 25), SCI (n = 24) and SCI + Hi1a (n = 27). (**H**) Average decay time for naive (n = 25), SCI (n = 24) and SCI + Hi1a (n = 27). *P < 0.05 from one-way ANOVA with Tukey’s multiple comparisons.

The expression of specific voltage-gated ion channels underpins AP discharge patterns in DDH INs [21–23]. We used a specific voltage-clamp protocol that clearly identifies three major subthreshold currents: I_Ar_, I_As_ and I_Ca_. I_Ar_ and I_As_ were quantitatively distinguished by a sharp peak (I_AR_) versus a sloping peak (I_AS_) (Fig 4A) [42]. In some neurons (22–29% across experimental groups), more than one ‘dominant’ subthreshold current existed, typically combinations of I_As_ and I_Ca_. These were classified as ‘mixed’ currents. The protocol we use to isolate subthreshold currents (Fig 5A) first hyperpolarises the neuron to –90 mV (1 s duration), from a holding potential of –60 mV (the average RMP for recorded DDH INs across groups was –62.2 mV; Fig 2B). This is followed by a depolarising step to –40 mV (200 ms duration), before returning to the –60 mV holding potential. It is important to note that the value of –40 mV is typically subthreshold for APs in DDH INs (Fig 2D); nonetheless, in some cases currents could not be resolved due to AP contamination.

Subthreshold currents were clearly resolved in 9 out of 14 (64%) in the naive group, 8 of 15 neurons (53%) in the SCI group and 14 out of 21 (67%) in the SCI + Hi1a group (Fig 4). Interestingly, we were unable to resolve any I_Ar_ currents from DDH INs in the SCI group. A two-tailed Fisher’s exact test showed no significant differences in the incidence of any of the subthreshold currents across the experimental groups however, as reported for AP discharge patterns, some reorganisation of subthreshold current expression was nonetheless observed across the treatment groups. This consistency in the redistribution of discharge categories and causal subthreshold currents further reflects the heterogeneity of the population of recorded interneurons in the DDH, and the subtle adaptations this diverse group of INs expresses after SCI or treatment with Hi1a.

### Spontaneous excitatory synaptic transmission

To assess whether acute inhibition of ASIC1a channels has a lasting effect on the presynaptic network or postsynaptic neuronal excitability, we recorded sEPSCs from DDH INs across the three experimental groups. Under our recording conditions (holding current of –60 mV, K^+^-gluconate internal solution) sEPSCs appear as inward currents (Fig 5A–5C) and have previously been shown to be mediated via the activation of AMPA-type glutamate receptors [28], because they are abolished by 10 μM CNQX [28, 55].

No significant differences in the frequency of EPSCs across naive, SCI and SCI + Hi1a groups were revealed via Kruskal–Wallis one-way ANOVA with Dunn’s multiple comparison analyses indicating that neither SCI nor acute treatment with Hi1a have meaningful effects on *presynaptic* network excitability in the DDH.

However, pronounced changes were observed in many EPSCs kinetic parameters. Individual sEPSCs for each recorded DDH IN were averaged and the peak amplitude (baseline to peak negative current), rise time (10–90% of peak amplitude), half-width (measured at 50% of peak amplitude) and decay time constant (20–80% of the decay phase) were determined (Fig 5A–5C). A significantly decreased rise time, half-width and decay time constant for sEPSCs in SCI + Hi1a DDH INs compared to naive INs (Fig 5F–5H) was revealed via analyses by one-way ANOVA with Tukey multiple comparisons or Kruskal–Wallis one-way ANOVA with Dunn’s multiple comparisons. However, there was no significance between naive and SCI, nor SCI and SCI + Hi1a groups in these same parameters, despite the apparent difference in data spread of sEPSC rise time represented graphically in Fig 5F (mean rise time ± SD: naive 1.27 ± 0.43 ms; SCI 0.98 ± 0.20 ms and SCI + Hi1a 1.02 ± 0.31 ms).

Peak amplitude was not different between any groups (Kruskal–Wallis one-way ANOVA with Dunn’s multiple comparisons). Overall, this indicates that sEPSC kinetics have reduced temporal components long-term after SCI when animals are treated with Hi1a. This reflects changes in specific excitatory receptor (AMPA-type glutamate) kinetics, or the somatodendritic location of glutamatergic receptors, that manifest in the changed temporal components of sEPSCs.

**Fig 6:**
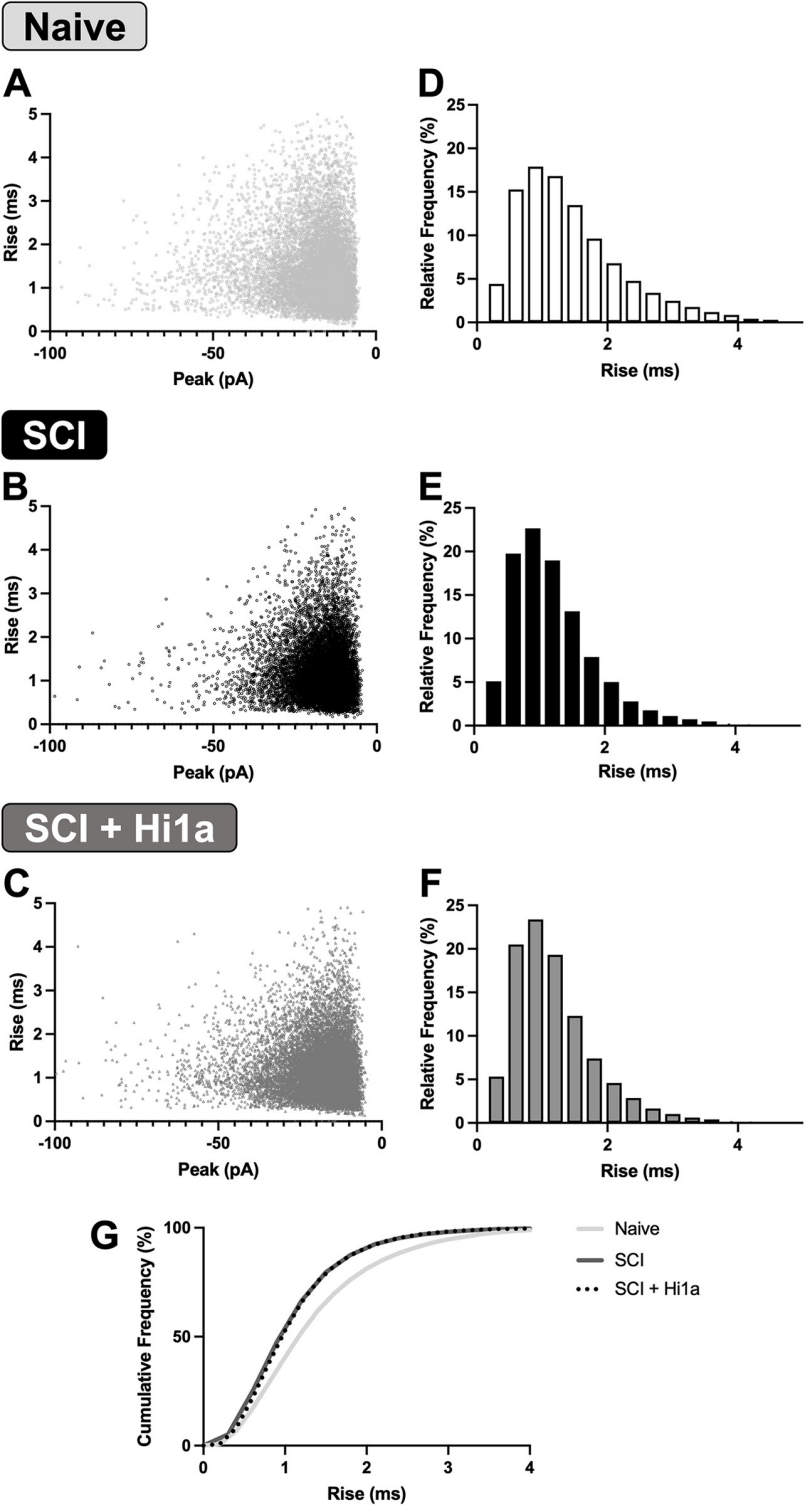
Lack of electrotonic filtering in naive, SCI and SCI + Hi1a mice. (**A–C**) Scatterplots of average sEPSC peak amplitude vs rise time in naive (light grey, n total sEPSCs = 10,505 sEPSCs), SCI (black, n = 11,902) and SCI + Hi1a (dark grey, n = 14,646) mice. (**D–F**) Relative frequency of sEPSC rise times in naive (light grey, n = 10,505), SCI (black, n = 11,902) and SCI + Hi1a (dark grey, n = 14,646) mice. (**G**) Cumulative frequency (%) of sEPSC rise times in naive (light grey), SCI (dark grey) and SCI + Hi1a (dashed line) mice.

The altered rise time, half-width and decay time constants of sEPSCs long-term after SCI has been reported to result from changes in glutamatergic receptor kinetics, synapse location on a the somatodendritic tree [56], or changes in dendritic architecture [57]. To further probe whether acute Hi1a treatment alters glutamatergic receptor kinetics or changes the synaptic or dendritic architecture of DDH INs, we assessed the relationship between sEPSC amplitude and rise time. To visualise these findings, peak sEPSC amplitude was plotted against rise time for naive (10,505 sEPSCs), SCI (11,902 sEPSCs) and SCI + Hi1a groups (14,646 sEPSCs). Overall, the data visualisation shows little variability in rise time across the three experimental groups (Fig 6A–6C). Indeed, the relative frequency of specific rise time values was plotted for sEPSCs from all experimental groups (Fig 6D–6F) and there were no significant differences in the relative frequency of binned rise times (two-way ANOVA, Tukey’s multiple comparison test). In the cumulative frequency plots of rise times (Fig 6G), a clear leftward shift in the cumulative frequency plot is evident in the SCI and SCI + Hi1a groups versus the naive mice. When using two-way ANOVA, the main column effect revealed the SCI and SCI + Hi1a groups to be significantly different from naive (*P* < 0.0001). However, when using row comparisons, this shift was only significant between the SCI + Hi1a and naive experimental groups for events with shorter duration rise times (< 3.6ms; two-way ANOVA, Tukey’s multiple comparison test). This is consistent with the faster rise times in sEPSCs recorded from INs from the SCI + Hi1a group (Fig 5F) and further reinforces that these changes are prevalent only for excitatory events with shorter rise times in Hi1a-treated mice.

Together these analyses show an absence of significant electrotonic filtering in DDH IN networks for SCI mice, both with and without Hi1a treatment, compared to naive mice. Thus, a substantive alteration in synapse location or architecture of the somatodendritic arbor of DDH INs is unlikely. The faster sEPSC kinetics in both SCI experimental groups therefore suggest that changes in glutamatergic receptor kinetics occur in DDH INs after SCI.

## Discussion

In this study we administered a single intravenous dose of Hi1a, a potent venom-derived inhibitor of ASIC1a, to assess long-term effects of acute ASIC1a inhibition on intrinsic membrane properties and excitatory synaptic transmission after SCI in adult mice. We used whole-cell patch-clamp electrophysiology in a horizontal spinal cord slice preparation to record intrinsic and synaptic properties of INs in the DDH that play a key role in relaying sensory information to downstream motor neurons. The spinal cord slice preparation used in this study is based on an extensive body of research showing that DDH INs communicate with the corticospinal tract, and contribute to plasticity post-SCI [17, 21–24, 28, 55, 58]. Spinal cord neuronal populations naturally exhibit morphological and physiological heterogeneity. The population of DDH INs we recorded from possess axons that do not leave the spinal cord and are comprised of a heterogeneous population of long or short propriospinal neurons and commissural neurons [59]. Neuron identity was not defined in our recordings, and thus data are a random sample from the DDH IN population.

Here, we summarise two key findings: (1) both SCI and SCI + Hi1a treatment preserved passive intrinsic or specific voltage-gated channel properties and (2) significant changes to sEPSCs properties were only observed in Hi1a-treated SCI mice when compared to naive animals. This highlights the selective effects of acute ASIC1a inhibition on specific ligand-gated receptor channels (i.e., glutamatergic receptors that mediate EPSCs) long-term after SCI, but not on other voltage-activated channels such as outward K^+^ channels that mediate subthreshold voltage-activated currents.

### 1. No changes to passive intrinsic or specific voltage-gated channel properties

Firstly, DDH INs from SCI and SCI + Hi1a treated animals did not exhibit lasting changes to passive or active intrinsic membrane properties at 4-weeks post injury. The proportion of AP discharge patterns between experimental groups were not significantly different (Fig 3B–3D), nor were there any detectable differences between experimental groups in the proportion of voltage-gated subthreshold currents that are known to underlie the specific AP discharge categories (Fig 5B–5D). When we applied a specific voltage protocol to elicit and compare select voltage-gated subthreshold currents, we were unable to detect any significant differences in the prevalence of I_Ar_, I_As_ or I_Ca_ currents across the experimental groups. However, an observable, but not statistically detectable, reorganisation of the proportion of both AP discharge patterns and voltage-gated subthreshold currents was present. Collectively these data reflect the diversity of the responses to SCI and SCI + Hi1a treatment from the heterogeneous population of DDH INs we recorded from and, moreover, indicate that SCI and/or acute treatment with Hi1a does not exert significant long-term effects on the specific voltage-activated channels that underlie intrinsic membrane, AP firing, or AHP properties.

### 2. sEPSC kinetics are altered in Hi1a-treated mice

sEPSCs are a measure of network and neuronal excitability. ASIC1a is necessary for motor learning by regulating plastic responses in spiny neurons, involved in N-methyl-D-aspartate receptor (NMDAR) mediated long-term depression in the hippocampus, associated with AMPAR trafficking evoking long-term synaptic potentiation, and involved in modulating neuronal transmission in the mouse Calyx of Held [60–63]. Increased presynaptic vesicle release is seen in hippocampal neurons cultured from ASIC1a knockout mice, suggesting that ASIC1a is expressed presynaptically [64]. Yet postsynaptic ASIC1a-dependent currents have been detected during synaptic transmission in the medial nucleus of trapezoid body neurons [65]. The prevailing evidence therefore suggests that ASIC1a is in some way involved in synaptic transmission, either pre- or post-synaptically (or both).

In our study, sEPSC kinetics (half-width, rise time, and decay time) were significantly decreased in Hi1a-treated SCI mice versus naive animals, indicating that DDH INs from Hi1a-treated SCI mice have decreased temporal components and faster kinetics at the post-synaptic membrane (more so than with SCI alone). As EPSCs in the horizontal spinal cord slice model and under the specific recording conditions used here have previously been shown to be mediated via the activation of AMPA-type glutamate receptors [28], our data suggest that acute inhibition of ASIC1a has long-term postsynaptic effects on excitatory synaptic transmission. We postulate a number of factors to explain this. Individual sEPSC kinetics could be faster due to: (i) increased presynaptic excitatory synaptic drive; (ii) synapse location on a neuron’s somatodendritic tree/changes in dendritic architecture [56, 57]; and (iii) changes in postsynaptic glutamatergic receptor subunits, distribution, or both. It is prudent to keep in mind that this study was performed at 4 weeks post-Hi1a administration, and these changes are driven by Hi1a intervention at a crucial time (1 h) post-SCI when tissue acidification in the peri-injury region—as well as ASIC1a expression—is substantively increased (Hu et al., 2011). In addition, we note that the data clusters for SCI versus naive animals for rise time exhibit a similar data spread to SCI + Hi1a; given the sample size and values shown, the potential of a Type II error in these analyses should be considered.

### (i) Excitatory synaptic drive or increased excitatory INs unlikely

It was previously shown ASIC1a expression is increased on *inhibitory* GABAergic INs compared to excitatory INs [66, 67]. A reduction in glycinergic neurotransmission suggests a loss of inhibitory drive in radial neurons after partial sciatic nerve ligation [68]. Therefore, our data may relate to a preferential loss of inhibitory INs, leading to a loss of inhibitory modulation and thus increased excitatory synaptic drive or an increased number of excitatory INs. However, we see no significant difference of AP thresholds between groups (despite a more hyperpolarised AP threshold in Hi1a-treated mice), nor in sEPSC frequency or between firing patterns to suggest changes to excitatory phenotypes [51, 69–71].

### (ii) Increased complexity of dendritic arbour unlikely

Electrotonic filtering, the phenomenon of attenuated synaptic potentials from more distal input—resulting in slower currents—reflects the distance of the recorded current from the soma [72]. Assuming space clamping was similar in all preparations, one explanation for the faster sEPSC kinetics we observed is that sEPSCs may arise from different/altered sites on the dendritic trees of DDH INs during the recovery process. Such reorganisation would change the extent of electrotonic filtering for sEPSCs recorded at the soma [73, 74]. To probe whether acute Hi1a treatment alters the synaptic location of sEPSCs in DDH INs, we analysed the relationship between sEPSC amplitude and rise time as a measure of electrotonic filtering (Fig 6). These analyses revealed no changes in the variability of rise times across SCI and SCI + Hi1a experimental groups, although sEPSCs from both of these groups did show *faster* rise times compared to INs from naive mice. An increase in the complexity of the dendritic arbour, or in the distance of the synaptic input from the soma of the recorded INs, would more likely result in *slower* sEPSC kinetics (reduced rise times and amplitudes). Our data do not support the presence of increased electrotonic filtering, but instead point to the possibility that sEPSC inputs after SCI may relocate closer to the soma, regardless of Hi1a-treatment. Our findings are consistent with previous reports of reduced dendritic branching that occurs in the early phases of recovery from SCI [57, 75–77]. Such reduced dendritic complexity would reduce the incidence of synapses located at long distances from the soma and contribute to the altered electrotonic filtering we observed in DDH INs [78, 79]. Our data are likewise consistent with similar studies performed by our group that also observed altered electrotonic filtering in DDH INs 3–4 weeks after incomplete SCI [21].

### (iii) Glutamatergic receptor distribution or subunit composition

sEPSCs expressed by DDH INs from Hi1a-treated SCI mice exhibited reduced rise times, half-width and decay time constants, which altogether indicate faster kinetics. Under our recording conditions, sEPSCs are mediated via the activation of AMPA-type glutamate receptors because they are completely abolished in the majority of neurons by 10 μM CNQX [28].

Mango and Nisticò [60] found that, in the hippocampus, ASIC1a contributes to peak amplitude of NMDAR- and AMPAR/kainate-driven EPSCs. Interestingly, *ASIC1* gene deletion was shown to alter the AMPAR:NDMAR ratio differentially depending on the neuronal population [4, 64, 80]. Other overlaps between these two recepors include protein-interacting with C kinase (PICK1) which binds to, and modulates the expression of, both ASIC1a and AMPAR on the cell surface [81, 82]. AMPAR phosphorylation can alter plasticity by enhancing channel conductance (e.g., permeability to ions) or time spent open, depending upon the site targeted, and this is a direct contributor to long-term potentiation (LTP) [83–85]. Glutamate-induced LTP has been previously shown to enhance sEPSC amplitude (but not frequency) [86].

Importantly, an increase in phosphorylation in the postsynaptic membrane, specifically on the AMPA-GluA1 subunit, is inhibited by PcTx1, a potent ASIC1a inhibitor, which led to the suggestion that ASIC1a modulates both intrinsic excitability and synaptic plasticity [87]. This is further supported by the observation that ASIC1a leads to phosphorylation of protein kinase C lambda (PKCλ), which in turn phosphorylates GluA1, thus providing a link between ASIC1a and AMPAR-involved LTP [61, 88].

As the sEPSCs from INs in the Hi1a-treated cohort have faster kinetics, this may result from AMPAR alteration (e.g., phosphorylation) in neurons [89, 90]. It is not known whether there was a redistribution of the AMPAR:NDMA ratio in our spinal cord preparation, or a change to the AMPAR subunit composition on DDH INs, but such future investigations will form a necessary part of studies to further inform the field.

## Conclusions

A single moderate dose of Hi1a administered within 1 h of SCI, and not SCI alone, results in significant alterations to the kinetics of sEPSCs of DDH INs when compared to naive animals. Therefore, our study suggests, using high-fidelity readouts of functional neurophysiological data, that acute inhibition of ASIC1a has long-term effects on sEPSC kinetics after SCI, potentially mediated by AMPAR plasticity. Acute inhibition of ASIC1a after SCI does not, however, exert any detectable long-term effects on AP discharge properties or the prevalence of specific voltage-gated subthreshold currents that underlie AP discharge types.

## Supporting information

S1 Fig Experimental timeline and procedure. (**A**) Timeline for patch clamp electrophysiology experiments showing all interventions and drug administration. Experiment begins at spinal cord injury surgery (0 h). Mice are anaesthetized via inhaled isoflurane anaesthesia (5% in O_2_ induction dose; 1–2% in O_2_ maintenance dose) and administered analgesics before surgical procedures are begun (subcutaneous (s.c.), buprenorphine (0.1 mg/kg) and carprofen (5 mg/kg)). At 1 h after surgery, the ASIC1a inhibitor Hi1a is administered via tail vein injection (i.v. 25 μg). Maintenance analgesics were given to all mice after surgery, continuously (transdermal Fentanyl patch, 12 mcg/h, 72 h) and at 12 h intervals (carprofen 5 mg/kg s.c.) for 2 d with hydration support provided via saline injections at 12 h intervals for 2 d (0.9% NaCl s.c.). At 3 d post-surgery, fentanyl patches were removed, and mice were returned to their home cage groups (naive, n = 36; SCI, n = 17; SCI + Hi1a, n = 14). At four weeks post SCI surgery, mice were humanely euthanised using ketamine (i.p. 100 mg/kg). Spinal cords were then dissected and prepared for patch clamp electrophysiology (image from BioRender) experiments as described. (**B**) Schematic of mouse spinal cord showing dorsal column spinal cord slice preparation. The preparation allows patching from INs in the DDH that are ideally placed to maintain connections between the sensory and motor pathways in the dorsal columns. Modified from Rank, Flynn [21].
(TIFF)

S1 Table Statistical results and effect sizes for intrinsic passive (RMP, I_R_, rheobase) and active membrane properties (AP threshold, AP peak, AP width, AHP peak). ANOVA^(A)^ Tukey’s/Kruskal Wallis ^(K)^ Dunn’s multiple comparisons tests, dependant on data normality under Shapiro-Wilk and Kolmogorov-Smirnov. No statistically significant differences between cohorts. Significance set at P < 0.005.
(PDF)

S2 Table Statistical analyses of sEPSC frequency and kinetics. Kruskal Wallis Dunn’s Multiple comparisons. Significance set at P < 0.005.
(PDF)

S3 Table Statistical analyses of cell location comparisons in SCI mice. ANOVA^(A)^ Tukey’s/Kruskal Wallis ^(K)^ Dunn’s multiple comparisons tests, dependant on data normality under Shapiro-Wilk and Kolmogorov-Smirnov. Contralateral caudal (n = 3), contralateral rostral (n = 7), ipsilateral caudal (n = 8), ipsilateral rostral (n = 2) and contralateral to epicentre (n = 2). Neuron location has no statistically significant effect on membrane properties. Significance set at P < 0.005.
(PDF)

S4 Table Statistical analyses of cell location comparisons in SCI mice treated with Hi1a. ANOVA^(A)^ Tukey’s/Kruskal Wallis ^(K)^ Dunn’s multiple comparisons tests, dependant on data normality under Shapiro-Wilk and Kolmogorov-Smirnov. Contralateral caudal (n = 4), contralateral rostral (n = 11), ipsilateral caudal (n = 4), ipsilateral rostral (n = 2) and contralateral to epicentre (n = 2). Significance is seen for sEPSC frequency between contralateral caudal and contralateral rostral regions of the spinal cord, but in no other parameters. Significance set at P < 0.005.
(PDF)

S5 Table Simple linear analyses for inclusion of access resistance above 20 Ohms. Spearman’s r correlation statistics p-values. Significance set at P < 0.005.
(PDF)

S6 Table Simple linear analyses for RMP effects on AP characteristics. Spearman’s r correlation statistics p-values. Significance set at P < 0.005.
(PDF)

## References

[1] Moskowitz M, Lo E, Iadecola C. The science of stroke: mechanisms in search of treatments. Neuron. 2010;67(2):181–98. doi: 10.1016/j.neuron.2010.07.002 20670828 PMC2957363

[2] Waldmann R, Champigny G, Bassilana F, Heurteaux C, Lazdunski M. A proton-gated cation channel involved in acid-sensing. Nature. 1997;386(6621):173. doi: 10.1038/386173a0 9062189

[3] Noël J, Salinas M, Baron A, Diochot S, Deval E, Lingueglia E. Current perspectives on acid-sensing ion channels: new advances and therapeutic implications. Expert Review of Clinical Pharmacology. 2010;3(3):331–46. doi: 10.1586/ecp.10.13 22111614

[4] Wemmie JA, Chen J, Askwith CC, Hruska-Hageman AM, Price MP, Nolan BC, et al. The acid-activated ion channel ASIC contributes to synaptic plasticity, learning, and memory. Neuron. 2002;34(3):463–77. doi: 10.1016/s0896-6273(02)00661-x 11988176

[5] Wemmie J, Askwith C, Lamani E, Cassell MD, Freeman J, Welsh M. Acid-sensing ion channel 1 is localized in brain regions with high synaptic density and contributes to fear conditioning. J Neurosci. 2003;23(13):5496–502. doi: 10.1523/JNEUROSCI.23-13-05496.2003 12843249 PMC6741257

[6] Krishtal OA, Osipchuk YV, Shelest TN, Smirnoff SV. Rapid extracellular pH transients related to synaptic transmission in rat hippocampal slices. Brain Research. 1987;436(2):352–6. doi: 10.1016/0006-8993(87)91678-7 2829992

[7] Foster VS, Rash LD, King GF, Rank MM. Acid-sensing ion channels: expression and function in resident and infiltrating immune cells in the central nervous system. Frontiers in Cellular Neuroscience. 2021;15:376. doi: 10.3389/fncel.2021.738043 34602982 PMC8484650

[8] Wang Y-Z, Wang J-J, Huang Y, Liu F, Zeng W-Z, Li Y, et al. Tissue acidosis induces neuronal necroptosis via ASIC1a channel independent of its ionic conduction. eLife. 2015;4. doi: 10.7554/eLife.05682 26523449 PMC4629285

[9] Hu R, Duan B, Wang DS, Yu Y, Li WG, Luo HS, et al. Role of acid-sensing ion channel 1a in the secondary damage of traumatic spinal cord injury. Ann Surg. 2011;254(2):353–62. doi: 10.1097/SLA.0b013e31822645b4 21725232

[10] Koehn LM, Noor NM, Dong Q, Er S-Y, Rash LD, King GF, et al. Selective inhibition of ASIC1a confers functional and morphological neuroprotection following traumatic spinal cord injury. F1000Research. 2016;5. doi: 10.12688/f1000research.9094.2 28105306 PMC5200949

[11] Wang J-J, Liu F, Yang F, Wang Y-Z, Qi X, Li Y, et al. Disruption of auto-inhibition underlies conformational signaling of ASIC1a to induce neuronal necroptosis. Nat Commun. 2020;11(1):475. doi: 10.1038/s41467-019-13873-0 31980622 PMC6981194

[12] Chassagnon IR, McCarthy CA, Chin YK, Pineda SS, Keramidas A, Mobli M, et al. Potent neuroprotection after stroke afforded by a double-knot spider-venom peptide that inhibits acid-sensing ion channel 1a. Proc Natl Acad Sci U S A. 2017;114(14):3750–5. doi: 10.1073/pnas.1614728114 28320941 PMC5389327

[13] Pignataro G, Simon RP, Xiong Z-G. Prolonged activation of ASIC1a and the time window for neuroprotection in cerebral ischaemia. Brain. 2007;130(1):151–8. doi: 10.1093/brain/awl325 17114797

[14] McCarthy CA, Rash LD, Chassagnon IR, King GF, Widdop RE. PcTx1 affords neuroprotection in a conscious model of stroke in hypertensive rats via selective inhibition of ASIC1a. Neuropharmacology. 2015;99:650–7. doi: 10.1016/j.neuropharm.2015.08.040 26320544

[15] Mazzone GL, Veeraraghavan P, Gonzalez-Inchauspe C, Nistri A, Uchitel OD. ASIC channel inhibition enhances excitotoxic neuronal death in an in vitro model of spinal cord injury. Neuroscience. 2017;343:398–410. doi: 10.1016/j.neuroscience.2016.12.008 28003157

[16] Mango D, Nisticò R. Role of ASIC1a in normal and pathological synaptic plasticity. In: Pedersen SHF, editor. Reviews of Physiology, Biochemistry and Pharmacology. Cham: Springer International Publishing; 2020. p. 83–100. 10.1007/112_2020_45 32789788

[17] Tracey D. Ascending and descending pathways in the spinal cord. In: Paxinos G, editor. The Rat Nervous System (Third Edition). Burlington: Academic Press; 2004. p. 149–64.

[18] Morisset V, Nagy F. Nociceptive integration in the rat spinal cord: role of non-linear membrane properties of deep dorsal horn neurons. Eur J Neurosci. 1998;10(12):3642–52. doi: 10.1046/j.1460-9568.1998.00370.x 9875343

[19] Landry M, Nagy F. GABA(B) receptors and sensitization to pain. Journal de la Société de Biologie. 2009;203:87–97. 19358814 10.1051/jbio:2009009

[20] Galea MP, Darian-Smith I. Multiple corticospinal neuron populations in the macaque monkey are specified by their unique cortical origins, spinal terminations, and connections. Cerebral Cortex. 1994;4(2):166–94. doi: 10.1093/cercor/4.2.166 8038567

[21] Rank MM, Flynn JR, Galea MP, Callister R, Callister RJ. Electrophysiological characterization of spontaneous recovery in deep dorsal horn interneurons after incomplete spinal cord injury. Experimental Neurology. 2015b;271:468–78. doi: 10.1016/j.expneurol.2015.07.002 26177044

[22] Flynn JR, Dunn LR, Galea MP, Callister R, Callister RJ, Rank MM. Exercise training after spinal cord injury selectively alters synaptic properties in neurons in adult mouse spinal cord. J Neurotrauma. 2013;30(10):891–6. doi: 10.1089/neu.2012.2714 23320512 PMC3660076

[23] Rank MM, Flynn JR, Battistuzzo CR, Galea MP, Callister R, Callister RJ. Functional changes in deep dorsal horn interneurons following spinal cord injury are enhanced with different durations of exercise training. The Journal of Physiology. 2015a;593(1):331–45. doi: 10.1113/jphysiol.2014.282640 25556804 PMC4293071

[24] Rank MM, Galea MP, Callister R, Callister RJ. Is more always better? How different ‘doses’ of exercise after incomplete spinal cord injury affects the membrane properties of deep dorsal horn interneurons. Experimental Neurology. 2018;300:201–11. doi: 10.1016/j.expneurol.2017.11.007 29146456

[25] Battistuzzo CR, Rank MM, Flynn JR, Morgan DL, Callister R, Callister RJ, et al. Gait recovery following spinal cord injury in mice: Limited effect of treadmill training. J Spinal Cord Med. 2016;39(3):335–43. doi: 10.1080/10790268.2015.1133017 26781526 PMC5073763

[26] Battistuzzo CR, Rank MM, Flynn JR, Morgan DL, Callister R, Callister RJ, et al. Effects of treadmill training on hindlimb muscles of spinal cord–injured mice. Muscle & Nerve. 2017;55(2):232–42. doi: 10.1002/mus.25211 27273462 PMC5324672

[27] Faul F, Erdfelder E, Buchner A, Lang AG. Statistical power analyses using G*Power 3.1: tests for correlation and regression analyses. Behaviour Research Methods. 2009;41(4):1149–60 doi: 10.3758/BRM.41.4.1149 19897823

[28] Flynn JR, Brichta AM, Galea MP, Callister RJ, Graham BA. A horizontal slice preparation for examining the functional connectivity of dorsal column fibres in mouse spinal cord. Journal of Neuroscience Methods. 2011b;200(2):113–20. doi: 10.1016/j.jneumeth.2011.06.017 21726580

[29] Graham BA, Brichta A, Schofield P, Callister R. Altered potassium channel function in the superficial dorsal horn of the spastic mouse. Journal of Physiology. 2007;584(1):121–36. doi: 10.1113/jphysiol.2007.138198 17690143 PMC2277054

[30] Graham BA, Brichta AM, Callister RJ. Recording temperature affects the excitability of mouse superficial dorsal horn neurons, in vitro. Journal of Neurophysiology. 2008;99(5):2048–59. doi: 10.1152/jn.01176.2007 18287548

[31] Smith KM, Boyle KA, Mustapa M, Jobling P, Callister RJ, Hughes DI, et al. Distinct forms of synaptic inhibition and neuromodulation regulate calretinin-positive neuron excitability in the spinal cord dorsal horn. Neuroscience. 2016;326:10–21. doi: 10.1016/j.neuroscience.2016.03.058 27045594 PMC4919388

[32] Yin JB, Lu YC, Feng B, Wu ZY, Chen YB, Zhang T, et al. Endomorphin-2 inhibits the activity of the spinoparabrachial projection neuron through presynaptic mechanisms in the spinal dorsal horn in rats. Neurosignals. 2018;26(1):43–57. doi: 10.1159/000488275 29554653

[33] Barry PH, Lynch JW. Liquid junction potentials and small cell effects in patch-clamp analysis. The Journal of Membrane Biology. 1991;121(2):101–17. doi: 10.1007/BF01870526 1715403

[34] Molleman A. The practice of patch clamping. Patch Clamping2002. p. 95–114.

[35] Tadros MA, Harris BM, Anderson WB, Brichta AM, Graham BA, Callister RJ. Are all spinal segments equal: intrinsic membrane properties of superficial dorsal horn neurons in the developing and mature mouse spinal cord. Journal of Physiology. 2012;590(10):2409–25. doi: 10.1113/jphysiol.2012.227389 22351631 PMC3424761

[36] Grudt TJ, Perl ER. Correlations between neuronal morphology and electrophysiological features in the rodent superficial dorsal horn. Journal of Physiology. 2002;540(1):189–207. doi: 10.1113/jphysiol.2001.012890 11927679 PMC2290200

[37] Ruscheweyh R, Ikeda H, Heinke B, Sandkühler J. Distinctive membrane and discharge properties of rat spinal lamina I projection neurones in vitro. The Journal of Physiology. 2004;555(2):527–43. doi: 10.1113/jphysiol.2003.054049 14694142 PMC1664848

[38] Yoshimura M, Jessell TM. Primary afferent-evoked synaptic responses and slow potential generation in rat substantia gelatinosa neurons in vitro. Journal of Neurophysiology. 1989;62(1):96–108. doi: 10.1152/jn.1989.62.1.96 2754484

[39] Hughes DI, Sikander S, Kinnon CM, Boyle KA, Watanabe M, Callister RJ, et al. Morphological, neurochemical and electrophysiological features of parvalbumin-expressing cells: a likely source of axo-axonic inputs in the mouse spinal dorsal horn. The Journal of Physiology. 2012;590(16):3927–51. doi: 10.1113/jphysiol.2012.235655 22674718 PMC3476641

[40] Hughes DI, Boyle KA, Kinnon CM, Bilsland C, Quayle JA, Callister RJ, et al. HCN4 subunit expression in fast-spiking interneurons of the rat spinal cord and hippocampus. Neuroscience. 2013;237:7–18. doi: 10.1016/j.neuroscience.2013.01.028 23357121 PMC3620460

[41] Candelas M, Reynders A, Arango-Lievano M, Neumayer C, Fruquière A, Demes E, et al. Cav3.2 T-type calcium channels shape electrical firing in mouse Lamina II neurons. Sci Rep. 2019;9(1):3112. doi: 10.1038/s41598-019-39703-3 30816223 PMC6395820

[42] Walsh MA, Graham BA, Brichta AM, Callister RJ. Evidence for a critical period in the development of excitability and potassium currents in mouse lumbar superficial dorsal horn neurons. Journal of Neurophysiology. 2009;101(4):1800–12. doi: 10.1152/jn.90755.2008 19176612

[43] Baron A, Voilley N, Lazdunski M, Lingueglia E. Acid sensing ion channels in dorsal spinal cord neurons. J Neurosci. 2008;28(6):1498–508. doi: 10.1523/JNEUROSCI.4975-07.2008 18256271 PMC6671562

[44] Wu LJ, Duan B, Mei YD, Gao J, Chen JG, Zhuo M, et al. Characterization of acid-sensing ion channels in dorsal horn neurons of rat spinal cord. Journal of Biological Chemistry. 2004;279(42):43716–24. doi: 10.1074/jbc.M403557200 15302881

[45] Duan B, Wu LJ, Yu YQ, Ding Y, Jing L, Xu L, et al. Upregulation of acid-sensing ion channel ASIC1a in spinal dorsal horn neurons contributes to inflammatory pain hypersensitivity. J Neurosci. 2007;27(41):11139–48. doi: 10.1523/JNEUROSCI.3364-07.2007 17928456 PMC6672839

[46] Mazzuca M, Heurteaux C, Alloui A, Diochot S, Baron A, Voilley N, et al. A tarantula peptide against pain via ASIC1a channels and opioid mechanisms. Nat Neurosci. 2007;10(8):943–5. doi: 10.1038/nn1940 17632507

[47] Vergo S, Craner M, Etzensperger R, Attfield K, Friese M, Newcombe J, et al. Acid-sensing ion channel 1 is involved in both axonal injury and demyelination in multiple sclerosis and its animal model. Brain. 2011;134(2):571–84. doi: 10.1093/brain/awq337 21233144

[48] Schuhmacher L-N, Smith ESJ. Expression of acid-sensing ion channels and selection of reference genes in mouse and naked mole rat. Molecular Brain. 2016;9(1):97. doi: 10.1186/s13041-016-0279-2 27964758 PMC5154015

[49] Xu Y, Jiang Y-Q, Li C, He M, Rusyniak WG, Annamdevula N, et al. Human ASIC1a mediates stronger acid-induced responses as compared with mouse ASIC1a. FASEB Journal. 2018;32(7):3832–43. doi: 10.1096/fj.201701367R 29447005 PMC5998965

[50] Siegel AF. Multiple t tests: some practical considerations. TESOL Quarterly. 1990;24(4):773–5

[51] Ruscheweyh R, Sandkühler J. Lamina-specific membrane and discharge properties of rat spinal dorsal horn neurones in vitro. The Journal of Physiology. 2002;541(1):231–44. doi: 10.1113/jphysiol.2002.017756 12015432 PMC2290304

[52] Patel AX, Burdakov D. Mechanisms of gain control by voltage-gated channels in intrinsically-firing neurons. PLoS One. 2015;10(3):e0115431. doi: 10.1371/journal.pone.0115431 25816008 PMC4376733

[53] Salinas E, Sejnowski TJ. Book review: gain modulation in the central nervous system: where behavior, neurophysiology, and computation meet. The Neuroscientist. 2001;7(5):430–40. 11597102 10.1177/107385840100700512 PMC2887717

[54] Salinas E, Thier P. Gain modulation: a major computational principle of the central nervous system. Neuron. 2000;27(1):15–21. doi: 10.1016/s0896-6273(00)00004-0 10939327

[55] Flynn JR, Graham BA, Galea MP, Callister RJ. The role of propriospinal interneurons in recovery from spinal cord injury. Neuropharmacology. 2011a;60(5):809–22. doi: 10.1016/j.neuropharm.2011.01.016 21251920

[56] Klee M, Rall W. Computed potentials of cortically arranged populations of neurons. Journal of Neurophysiology. 1977;40(3):647–66. doi: 10.1152/jn.1977.40.3.647 874533

[57] Gazula VR, Roberts M, Luzzio C, Jawad AF, Kalb RG. Effects of limb exercise after spinal cord injury on motor neuron dendrite structure. Journal of Comparative Neurology. 2004;476(2):130–45. doi: 10.1002/cne.20204 15248194

[58] Szûcs P, Odeh F, Szokol K, Antal M. Neurons with distinctive firing patterns, morphology and distribution in laminae V–VII of the neonatal rat lumbar spinal cord. Eur J Neurosci. 2003;17(3):537–44. doi: 10.1046/j.1460-9568.2003.02484.x 12581171

[59] Saywell SA, Ford TW, Meehan CF, Todd AJ, Kirkwood PA. Electrophysiological and morphological characterization of propriospinal interneurons in the thoracic spinal cord. Journal of Neurophysiology. 2010;105(2):806–26. doi: 10.1152/jn.00738.2010 21106900 PMC3059177

[60] Mango D, Nisticò R. Acid-sensing ion channel 1a is involved in n-methyl d-aspartate receptor-dependent long-term depression in the hippocampus. Front Pharmacol. 2019;10:555. doi: 10.3389/fphar.2019.00555 31178731 PMC6537656

[61] Li H-S, Su X-Y, Song X-L, Qi X, Li Y, Wang R-Q, et al. Protein kinase c lambda mediates acid-sensing ion channel 1a-dependent cortical synaptic plasticity and pain hypersensitivity. The Journal of Neuroscience. 2019;39(29):5773. doi: 10.1523/JNEUROSCI.0213-19.2019 31101759 PMC6636072

[62] Yu Z, Wu YJ, Wang YZ, Liu DS, Song XL, Jiang Q, et al. The acid-sensing ion channel ASIC1a mediates striatal synapse remodeling and procedural motor learning. Science Signalling. 2018;11(542):eaar4481. doi: 10.1126/scisignal.aar4481 30087178 PMC6324561

[63] Gonzales EB, Kawate T, Gouaux E. Pore architecture and ion sites in acid-sensing ion channels and P2X receptors. Nature. 2009;460(7255):599–604. doi: 10.1038/nature08218 19641589 PMC2845979

[64] Cho J-H, Askwith C. Presynaptic release probability is increased in hippocampal neurons from ASIC1 knockout mice. Journal of Neurophysiology. 2008;99(2):426–41. doi: 10.1152/jn.00940.2007 18094106

[65] Gonzalez-Inchauspe C, Urbano F, Di Guilmi M, Uchitel O. Acid-sensing ion channels activated by evoked released protons modulate synaptic transmission at the mouse Calyx of Held synapse. Journal of Neuroscience 2017;37(10):2589–99. doi: 10.1523/JNEUROSCI.2566-16.2017 28159907 PMC6596635

[66] Ziemann AE, Schnizler MK, Albert GW, Severson MA, Howard MA, Welsh MJ, et al. Seizure termination by acidosis depends on ASIC1a. Nat Neurosci. 2008;11(7):816–22. doi: 10.1038/nn.2132 18536711 PMC2553357

[67] Weng JY, Lin YC, Lien CC. Cell type-specific expression of acid-sensing ion channels in hippocampal interneurons. The Journal of Neuroscience. 2010;30(19):6548–58. doi: 10.1523/JNEUROSCI.0582-10.2010 20463218 PMC6632567

[68] Imlach WL, Bhola RF, Mohammadi SA, Christie MJ. Glycinergic dysfunction in a subpopulation of dorsal horn interneurons in a rat model of neuropathic pain. Sci Rep. 2016;6(37104). 10.1038/srep37104 PMC5107903 27841371

[69] Prescott SA, Koninck YD. Four cell types with distinctive membrane properties and morphologies in lamina I of the spinal dorsal horn of the adult rat. The Journal of Physiology. 2002;539(3):817–36. doi: 10.1113/jphysiol.2001.013437 11897852 PMC2290183

[70] Prescott SA, Ratté S. Pain processing by spinal microcircuits: afferent combinatorics. Current Opinion in Neurobiology. 2012;22(4):631–9. doi: 10.1016/j.conb.2012.02.010 22409855 PMC3388176

[71] Ratté S, Lankarany M, Rho Y-A, Patterson A, Prescott SA. Subthreshold membrane currents confer distinct tuning properties that enable neurons to encode the integral or derivative of their input. Frontiers in Cellular Neuroscience. 2015(8):452. doi: 10.3389/fncel.2014.00452 25620913 PMC4288132

[72] Häusser M. Synaptic function: Dendritic democracy. Curr Biol. 2001;11(1):R10–R2. doi: 10.1016/s0960-9822(00)00034-8 11166188

[73] Callister RJ, Walmsley B. Amplitude and time course of evoked and spontaneous synaptic currents in rat submandibular ganglion cells. The Journal of Physiology. 1996;490(1):149–57. doi: 10.1113/jphysiol.1996.sp021132 8745284 PMC1158653

[74] Magee JC. Dendritic integration of excitatory synaptic input. Nature Reviews Neuroscience. 2000;1(3):181–90. doi: 10.1038/35044552 11257906

[75] Bernstein JJ, Wacker W, Standler N. Spinal motoneuron dendritic alteration after spinal cord hemisection in the rat. Experimental Neurology. 1984;83(3):548–54. doi: 10.1016/0014-4886(84)90122-5 6698157

[76] Bose P, Parmer R, Reier PJ, Thompson FJ. Morphological changes of the soleus motoneuron pool in chronic midthoracic contused rats. Experimental Neurology. 2005;191(1):13–23. doi: 10.1016/j.expneurol.2004.08.028 15589508

[77] Kitzman P. Alteration in axial motoneuronal morphology in the spinal cord injured spastic rat. Experimental Neurology. 2005;192(1):100–8. doi: 10.1016/j.expneurol.2004.10.021 15698623

[78] London M, Häusser M. Dendritic computation. Annual Review of Neuroscience. 2005;28(1):503–32. doi: 10.1146/annurev.neuro.28.061604.135703 16033324

[79] van Ooyen A, Duijnhouwer J, Remme MW, van Pelt J. The effect of dendritic topology on firing patterns in model neurons. Network. 2002;13(3):311–25. doi: 10.1088/0954-898x/13/3/304 12222816

[80] Kreple C, Lu Y, Taugher R, Schwager-Gutman A, Du J, Stump M, et al. Acid-sensing ion channels contribute to synaptic transmission and inhibit cocaine-evoked plasticity. Nat Neurosci. 2014;17(8):1083–91. doi: 10.1038/nn.3750 24952644 PMC4115047

[81] Joch M, Ase AR, Chen CXQ, Macdonald PA, Kontogiannea M, Corera AT, et al. Parkin-mediated monoubiquitination of the PDZ protein PICK1 regulates the activity of acid-sensing ion channels. Molecular Biology of the Cell. 2007;18(8):3105–18. doi: 10.1091/mbc.e05-11-1027 17553932 PMC1949385

[82] Xia J, Zhang X, Staudinger J, Huganir R. Clustering of AMPA receptors by the synaptic PDZ domain-containing protein PICK1. Neuron. 1999;22(1):179–87. doi: 10.1016/s0896-6273(00)80689-3 10027300

[83] Derkach V, Barria A, Soderling TR. Ca^2+^/calmodulin-kinase II enhances channel conductance of alpha-amino-3-hydroxy-5-methyl-4-isoxazolepropionate type glutamate receptors. Proc Natl Acad Sci U S A. 1999;96(6):3269–74. 10077673 10.1073/pnas.96.6.3269 PMC15931

[84] Banke TG, Bowie D, Lee H, Huganir RL, Schousboe A, Traynelis SF. Control of GluR1 AMPA receptor function by cAMP-dependent protein kinase. J Neurosci. 2000;20(1):89–102. doi: 10.1523/JNEUROSCI.20-01-00089.2000 10627585 PMC6774102

[85] Lee HK, Barbarosie M, Kameyama K, Bear MF, Huganir RL. Regulation of distinct AMPA receptor phosphorylation sites during bidirectional synaptic plasticity. Nature. 2000;405(6789):955–99. doi: 10.1038/35016089 10879537

[86] Cormier RJ, Kelly PT. Glutamate-induced long-term potentiation enhances spontaneous EPSC amplitude but not frequency. Journal of Neurophysiology. 1996;75(5):1909–18. doi: 10.1152/jn.1996.75.5.1909 8734590

[87] Mango D, Braksator E, Battaglia G, Marcelli S, Mercuri NB, Feligioni M, et al. Acid-sensing ion channel 1a is required for mGlu receptor dependent long-term depression in the hippocampus. Pharmacological Research. 2017;119:12–9. doi: 10.1016/j.phrs.2017.01.028 28137639

[88] Ren S-Q, Yan J-Z, Zhang X-Y, Bu Y-F, Pan W-W, Yao W, et al. PKCλ is critical in AMPA receptor phosphorylation and synaptic incorporation during LTP. EMBO J. 2013;32(10):1365–80. 23511975 10.1038/emboj.2013.60 PMC3655466

[89] Jenkins MA, Traynelis SF. PKC phosphorylates GluA1-Ser831 to enhance AMPA receptor conductance. Channels (Austin). 2012;6(1):60–4. doi: 10.4161/chan.18648 22373567 PMC3367675

[90] Quintana P, Soto D, Poirot O, Zonouzi M, Kellenberger S, Muller D, et al. Acid-sensing ion channel 1a drives AMPA receptor plasticity following ischaemia and acidosis in hippocampal CA1 neurons. The Journal of Physiology. 2015;593(19):4373–86. doi: 10.1113/JP270701 26174503 PMC4594240

