# Supplementary figures and images for: Acute inhibition of acid sensing ion channel 1a after spinal cord injury selectively affects excitatory synaptic transmission, but not intrinsic membrane properties, in deep dorsal horn interneurons
Source: PLoS One. 2023 Nov 8;18(11):e0289053. doi: 10.1371/journal.pone.0289053 (PMC10631665; doi:10.1371/journal.pone.0289053)

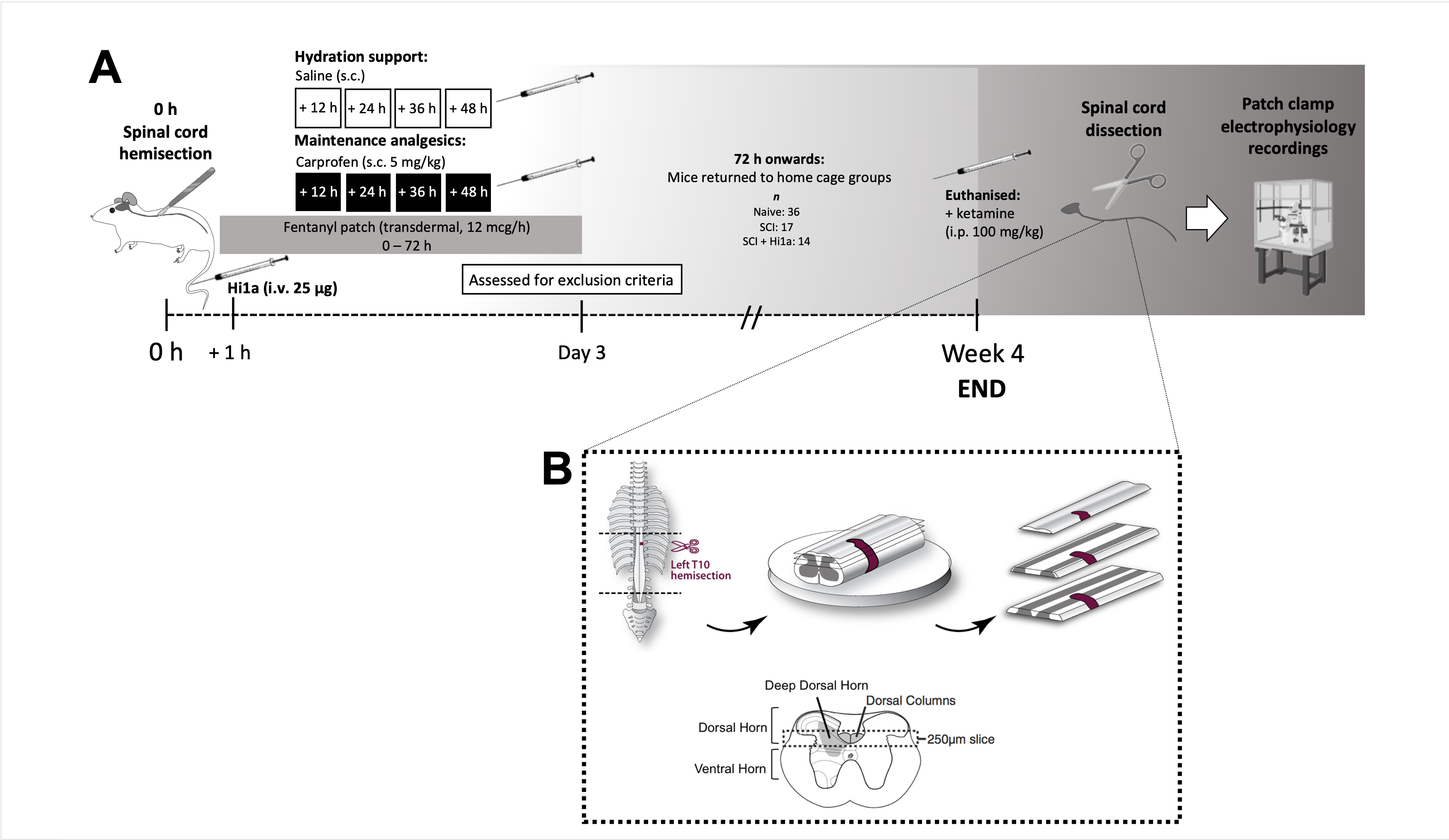

Supplement: S1 Fig — (A) Timeline for patch clamp electrophysiology experiments showing all interventions and drug administration. Experiment begins at spinal cord injury surgery (0 h). Mice are anaesthetized via inhaled isoflurane anaesthesia (5% in O2 induction dose; 1–2% in O2 maintenance dose) and administered analgesics before surgical procedures are begun (subcutaneous (s.c.), buprenorphine (0.1 mg/kg) and carprofen (5 mg/kg)). At 1 h after surgery, the ASIC1a inhibitor Hi1a is administered via tail vein injection (i.v. 25 μg). Maintenance analgesics were given to all mice after surgery, continuously (transdermal Fentanyl patch, 12 mcg/h, 72 h) and at 12 h intervals (carprofen 5 mg/kg s.c.) for 2 d with hydration support provided via saline injections at 12 h intervals for 2 d (0.9% NaCl s.c.). At 3 d post-surgery, fentanyl patches were removed, and mice were returned to their home cage groups (naive, n = 36; SCI, n = 17; SCI + Hi1a, n = 14). At four weeks post SCI surgery, mice were humanely euthanised using ketamine (i.p. 100 mg/kg). Spinal cords were then dissected and prepared for patch clamp electrophysiology (image from BioRender) experiments as described. (B) Schematic of mouse spinal cord showing dorsal column spinal cord slice preparation. The preparation allows patching from INs in the DDH that are ideally placed to maintain connections between the sensory and motor pathways in the dorsal columns. Modified from Rank, Flynn [21]. (TIFF) [file pone.0289053.s001.tiff]
