# Supplementary material for: Acute inhibition of acid sensing ion channel 1a after spinal cord injury selectively affects excitatory synaptic transmission, but not intrinsic membrane properties, in deep dorsal horn interneurons
Source: PLoS One. 2023 Nov 8;18(11):e0289053. doi: 10.1371/journal.pone.0289053 (PMC10631665; doi:10.1371/journal.pone.0289053)
Supplement: S1 Table — ANOVA(A) Tukey’s/Kruskal Wallis (K) Dunn’s multiple comparisons tests, dependant on data normality under Shapiro-Wilk and Kolmogorov-Smirnov. No statistically significant differences between cohorts. Significance set at P < 0.005. (PDF) [file pone.0289053.s002.pdf]

ANOVA (A) Tukey's/Kruskal Wallis (K) Dunn's multiple comparisons			
	Naive vs SCI	Naive vs Hi1a	SCI vs Hi1a
RMP [K]	>0.9999	0.5658	>0.9999
I_R [K]	>0.9999	0.9726	>0.9999
Rheobase [K]	>0.9999	>0.9999	>0.9999
AP Threshold [A]	0.6199	0.1663	0.7852
Peak [K]	>0.9999	0.8452	0.8879
Width [A]	0.7002	0.7219	0.9923
AHP Peak [A]	0.2948	0.4398	0.9158
