# Supplementary material for: Acute inhibition of acid sensing ion channel 1a after spinal cord injury selectively affects excitatory synaptic transmission, but not intrinsic membrane properties, in deep dorsal horn interneurons
Source: PLoS One. 2023 Nov 8;18(11):e0289053. doi: 10.1371/journal.pone.0289053 (PMC10631665; doi:10.1371/journal.pone.0289053)
Supplement: S2 Table — Kruskal Wallis Dunn’s Multiple comparisons. Significance set at P < 0.005. (PDF) [file pone.0289053.s003.pdf]

ANOVA (^{A}) Tukey's/Kruskal Wallis (^{K}) Dunn's multiple comparisons			
	Naive vs SCI	Naive vs Hi1a	SCI vs Hi1a
Frequency ^{K}	>0.9999	>0.9999	>0.9999
Peak ^{K}	>0.9999	0.1704	0.3010
Half-width^A	0.1499	0.0139	0.6190
Rise^K	0.1201	0.0390	>0.9999
Decay^A	0.2018	0.0257	0.5215
