# Supplementary material for: Acute inhibition of acid sensing ion channel 1a after spinal cord injury selectively affects excitatory synaptic transmission, but not intrinsic membrane properties, in deep dorsal horn interneurons
Source: PLoS One. 2023 Nov 8;18(11):e0289053. doi: 10.1371/journal.pone.0289053 (PMC10631665; doi:10.1371/journal.pone.0289053)
Supplement: S3 Table — ANOVA(A) Tukey’s/Kruskal Wallis (K) Dunn’s multiple comparisons tests, dependant on data normality under Shapiro-Wilk and Kolmogorov-Smirnov. Contralateral caudal (n = 3), contralateral rostral (n = 7), ipsilateral caudal (n = 8), ipsilateral rostral (n = 2) and contralateral to epicentre (n = 2). Neuron location has no statistically significant effect on membrane properties. Significance set at P < 0.005. (PDF) [file pone.0289053.s004.pdf]

	One-way ANOVA (^{A}) Tukey's/Kruskal Wallis (^{K}) Dunn's multiple comparisons for SCI cohort											
	$I_R^{(A)}$	RMP $^{(A)}$	sEPSC frequency $^{(K)}$	Rise $^{(A)}$	Peak $^{(A)}$	Decay $^{(A)}$	Half-width $^{(A)}$	AP firing $^{(A)}$	AP peak$^{(A)}$	AP thres-hold$^{(A)}$	AP rheo-base$^{(K)}$	AHP peak (A)
Contralateral caudal vs. Contralateral rostral	0.988	0.993	0.211	>0.999	0.607	0.993	>0.999	0.898	0.899	>0.999	>0.999	>0.999
Contralateral caudal vs. Ipsilateral caudal	0.880	0.986	0.186	0.997	0.744	0.729	0.772	0.859	0.945	0.954	>0.999	0.945
Contralateral caudal vs. Ipsilateral rostral	0.977	0.245	>0.999	0.973	0.785	>0.999	>0.999	>0.999	1.000	0.977	>0.999	0.977
Contralateral caudal vs. Contralateral to epicentre	0.850	0.918	>0.999	0.908	0.429	0.984	0.992	>0.999	0.598	0.709	>0.999	0.709
Contralateral rostral vs. Ipsilateral caudal	0.975	>0.999	>0.999	0.991	0.985	0.939	0.770	>0.999	0.999	0.904	>0.999	0.904
Contralateral rostral vs. Ipsilateral rostral	0.999	0.268	>0.999	0.985	1.000	0.998	>0.999	0.960	0.985	0.968	0.783	0.968
Contralateral rostral vs. Contralateral to epicentre	0.946	0.976	>0.999	0.936	0.988	1.000	0.991	0.960	0.870	0.6412	>0.999	0.641
Ipsilateral caudal vs. Ipsilateral rostral	>0.999	0.286	>0.999	0.868	0.999	0.850	0.845	0.952	0.995	>0.9999	>0.999	>0.999
Ipsilateral caudal vs. Contralateral to epicentre	0.996	0.984	>0.999	0.724	0.861	0.987	0.986	0.952	0.7995	0.8859	>0.999	0.886
Ipsilateral rostral vs. Contralateral to epicentre	0.995	0.747	>0.999	0.999	0.975	0.993	0.993	>0.999	0.7795	0.9675	>0.999	0.968
