# Supplementary material for: Acute inhibition of acid sensing ion channel 1a after spinal cord injury selectively affects excitatory synaptic transmission, but not intrinsic membrane properties, in deep dorsal horn interneurons
Source: PLoS One. 2023 Nov 8;18(11):e0289053. doi: 10.1371/journal.pone.0289053 (PMC10631665; doi:10.1371/journal.pone.0289053)
Supplement: S4 Table — ANOVA(A) Tukey’s/Kruskal Wallis (K) Dunn’s multiple comparisons tests, dependant on data normality under Shapiro-Wilk and Kolmogorov-Smirnov. Contralateral caudal (n = 4), contralateral rostral (n = 11), ipsilateral caudal (n = 4), ipsilateral rostral (n = 2) and contralateral to epicentre (n = 2). Significance is seen for sEPSC frequency between contralateral caudal and contralateral rostral regions of the spinal cord, but in no other parameters. Significance set at P < 0.005. (PDF) [file pone.0289053.s005.pdf]

	One-way ANOVA (^{A}) Tukey's/Kruskal Wallis (^{K}) Dunn's multiple comparisons for SCI + Hi1a cohort											
	$I_R^{(K)}$	RMP$^{(A)}$	sEPSC frequency $^{(K)}$	Rise$^{(A)}$	Peak $^{(A)}$	Decay$^{(A)}$	Half-width$^{(A)}$	AP firing$^{(A)}$	AP peak$^{(A)}$	AP thres-hold $^{(K)}$	AP rheo-base $^{(K)}$	AHP peak $^{(A)}$
Contralateral caudal vs. Contralateral rostral	>0.999	0.466	0.014	0.977	0.9964	0.707	0.601	0.596	0.906	>0.999	>0.999	0.848
Contralateral caudal vs. Ipsilateral caudal	>0.999	0.494	>0.999	0.200	0.7491	0.994	0.919	1.000	0.999	>0.999	>0.999	0.997
Contralateral caudal vs. Ipsilateral rostral	>0.999	0.993	>0.999	0.998	0.9964	0.977	>0.999	0.998	0.912	>0.999	>0.999	>0.999
Contralateral caudal vs. Contralateral to epicentre	>0.999	0.959	0.926	0.880	0.9964	0.972	0.835	0.928	>0.999	>0.999	>0.999	0.873
Contralateral rostral vs. Ipsilateral caudal	>0.999	1.000	0.356	0.236	0.2979	0.932	0.990	0.659	0.953	>0.999	>0.999	0.530
Contralateral rostral vs. Ipsilateral rostral	>0.999	0.952	0.426	>0.999	0.9839	0.997	0.833	0.698	0.639	>0.999	>0.999	0.990
Contralateral rostral vs. Contralateral to epicentre	>0.999	0.801	>0.999	0.971	0.9964	0.998	>0.999	0.966	0.927	>0.999	>0.999	0.208
Ipsilateral caudal vs. Ipsilateral rostral	>0.999	0.937	>0.999	0.557	0.9593	0.999	0.971	0.992	0.845	>0.999	>0.999	0.998
Ipsilateral caudal vs. Contralateral to epicentre	>0.999	0.802	>0.999	0.886	0.8411	0.999	0.996	0.966	>0.999	>0.999	>0.999	0.957
Ipsilateral rostral vs. Contralateral to epicentre	>0.999	>0.999	>0.999	0.983	0.9964	>0.999	0.912	0.912	0.881	>0.999	>0.999	0.956
