# Supplementary material for: Acute inhibition of acid sensing ion channel 1a after spinal cord injury selectively affects excitatory synaptic transmission, but not intrinsic membrane properties, in deep dorsal horn interneurons
Source: PLoS One. 2023 Nov 8;18(11):e0289053. doi: 10.1371/journal.pone.0289053 (PMC10631665; doi:10.1371/journal.pone.0289053)
Supplement: S5 Table — Spearman’s r correlation statistics p-values. Significance set at P < 0.005. (PDF) [file pone.0289053.s006.pdf]

	Naive	SCI	SCI + Hi1a
**RMP**	0.9866	0.8511	0.7542
**I_R**	0.2145	0.1499	0.7085
**sEPSC frequency**	0.3170	0.5639	0.4481
**sEPSC peak**	0.4254	0.0816	0.0786
**sEPSC rise**	0.1570	0.4577	0.7188
**sEPSC half-width**	0.3903	0.1446	0.8088
**sEPSC decay**	0.1545	0.6304	0.4925
