# Supplementary material for: Acute inhibition of acid sensing ion channel 1a after spinal cord injury selectively affects excitatory synaptic transmission, but not intrinsic membrane properties, in deep dorsal horn interneurons
Source: PLoS One. 2023 Nov 8;18(11):e0289053. doi: 10.1371/journal.pone.0289053 (PMC10631665; doi:10.1371/journal.pone.0289053)
Supplement: S6 Table — Spearman’s r correlation statistics p-values. Significance set at P < 0.005. (PDF) [file pone.0289053.s007.pdf]

| | Naive | SCI | SCI + Hi1a |
|---|---|---|---|
| **Rheobase** | 0.6654 | 0.5833 | 0.0916 |
| **Threshold** | 0.5611 | 0.3935 | 0.1407 |
| **AP peak** | 0.7024 | 0.2963 | 0.2679 |
| **AP width** | 0.3308 | 0.1701 | 0.3938 |
| **AHP peak** | 0.2629 | 0.1184 | 0.1017 |
